# Role of *Fasciola hepatica* Small RNAs in the Interaction With the Mammalian Host

**DOI:** 10.3389/fcimb.2021.812141

**Published:** 2022-01-20

**Authors:** Santiago Fontenla, Mauricio Langleib, Eduardo de la Torre-Escudero, Maria Fernanda Domínguez, Mark W. Robinson, José Tort

**Affiliations:** ^1^ Departamento de Genética, Facultad de Medicina, Universidad de la República (UdelaR), Montevideo, Uruguay; ^2^ Departamento de Desarrollo Biotecnológico, Instituto de Higiene, Facultad de Medicina, Universidad de la República (UdelaR), Montevideo, Uruguay; ^3^ School of Biological Sciences, Queen’s University Belfast, Belfast, Northern Ireland

**Keywords:** *Fasciola hepatica*, micro RNA (miRNA), development, tRNA, vault RNA (vtRNA), Extracellular vesicles (EVs), host-parasite

## Abstract

MicroRNAs (miRNAs) are important post-transcriptional regulators of gene expression being involved in many different biological processes and play a key role in developmental timing. Additionally, recent studies have shown that miRNAs released from parasites are capable of regulating the expression of host genes. In the present work, we studied the expression patterns of ncRNAs of various intra-mammalian life-cycle stages of the liver fluke, *Fasciola hepatica*, as well as those packaged into extracellular vesicles and shed by the adult fluke. The miRNA expression profile of the intra-mammalian stages shows important variations, despite a set of predominant miRNAs that are highly expressed across all stages. No substantial variations in miRNA expression between dormant and activated metacercariae were detected, suggesting that they might not be central players in regulating fluke gene expression during this crucial step in the invasion of the definitive host. We generated a curated pipeline for the prediction of putative target genes that reports only sites conserved between three different prediction approaches. This pipeline was tested against an iso-seq curated database of the 3’ UTR regions of *F. hepatica* genes to detect miRNA regulation networks within liver fluke. Several functions related to the host immune response or modulation were enriched among the targets of the most highly expressed parasite miRNAs, stressing that they might be key players during the establishment and maintenance of infection. Additionally, we detected fragments derived from the processing of tRNAs, in all developmental stages analyzed, and documented the presence of novel long tRNA fragments enriched in vesicles. We confirmed the presence of at least 5 putative vault RNAs (vtRNAs), that are expressed across different stages and enriched in vesicles. The presence of tRNA fragments and vtRNAs in vesicles raise the possibility that they could be involved in the host-parasite interaction.

## Introduction

Fasciolosis, or liver fluke disease, is caused by infection with two major liver fluke species: *Fasciola hepatica* in temperate regions of all continents and *Fasciola gigantica*, which is more restricted to tropical regions. Fasciolosis in ruminants is widespread and is responsible for massive economic losses to the livestock industry, estimated globally to be US$3.2 billion annually due to reduced production yields and associated treatment costs (Zerna et al., 2021). It is now also recognized as a neglected tropical disease of humans by the World Health Organization (WHO) (World Health Organization, 2007) with 17 million people being infected and another 180 million people at risk of acquiring the infection predominantly in developing countries. However, the actual numbers of infections in humans and animals are likely underestimated due to the lack of comprehensive or coordinated investigations, and limited availability of diagnostic tools in some developing countries (Harrington et al., 2017).

Liver flukes have complex life cycles. The definitive hosts, (either livestock or humans) acquire the infection by consuming water or plants (e.g., watercress) contaminated with parasite cysts (metacercariae). Gastric and duodenal contents promote excystation, and the newly excysted juveniles (NEJs) actively migrate across the intestinal wall into the peritoneal cavity, and then into the liver parenchyma. The final destination are the biliary ducts within the liver where they reach maturity, producing thousands of eggs that are released to the environment in the stool (Keiser and Utzinger, 2009).

Clinically, the acute phase of *F. hepatica* infection is characterized by marked eosinophilia, abdominal pain and fever. Beyond this acute form of the disease, a chronic stage once the parasite is established is characterized by intermittent obstruction of bile ducts, causing symptoms that resemble biliary colic and cholecystitis (Garcia et al., 2007). Like most helminths, *Fasciola* spp. have developed strategies to modulate the host immune response to promote their survival and reproduction. These play an essential role in the establishment of infection and are mediated by molecules secreted by the parasite and those of the host that activate repair mechanisms (Dalton et al., 2013).

While initial studies have focused largely on proteins as possible immunomodulators, the discovery of microRNAs that are secreted by the parasite revealed new mediators in the host-parasite interaction. MicroRNAs (miRNAs) are single-stranded non-coding 21 to 25 nucleotide long sequences, that negatively regulate the translation of messenger RNAs (mRNAs) by blockage of translation and/or mRNA destabilization (Bartel, 2009). MiRNAs were first described in the roundworm *Caenorhabditis elegans* with roles in the regulation of development (Lee et al., 1993; Reinhart et al., 2000). Since then, miRNAs have been described in all eukaryotes (Wheeler et al., 2009; Tarver et al., 2013) and perform varied functional roles including cellular differentiation, apoptosis, metabolism and silencing of transposable elements (Bushati and Cohen, 2007; Tristán-Ramos et al., 2020). MiRNAs have also been described in several platyhelminths (Palakodeti et al., 2006; Basika et al., 2016; Cai et al., 2016; Stroehlein et al., 2018), including the NEJ and adult stages of *F. hepatica* (Xu et al., 2012; Fontenla et al., 2015; Fromm et al., 2015) and more recently in eight stages of the life cycle of *F. gigantica* (Hu et al., 2021).

The secretion of EVs was initially described as a means of eliminating unneeded compounds from the cell (Johnstone et al., 1987) but later works reported the mRNA and miRNA cargo within the EVs of mammalian cells suggesting that they have roles in regulation of gene expression (Valadi et al., 2007; Mitchell et al., 2008). In helminths, miRNAs contained in EVs was first reported in the trematode *Dicrocoelium dendrticum*, soon followed by reports in the clade V nematode *Heligmosomoides polygyrus* (Bernal et al., 2014; Buck et al., 2014), and later by similar findings in several other nematode, trematode and cestode species (Fromm et al., 2015; Nowacki et al., 2015; Sotillo et al., 2020; Avni and Avni, 2021; Cucher et al., 2021).

Parasite-specific miRNAs have been detected in the blood of hosts infected with several nematodes including *Onchocerca ochengi* and *O. volvulus* (Quintana et al., 2015), *Loa* (Tritten et al., 2014b), *Dirofilaria immitis* (Tritten et al., 2014a), *Litomosoides sigmodontis* (Buck et al., 2014; Quintana et al., 2019) and trematodes such as schistosomes (Cheng et al., 2013; Hoy et al., 2014; Cai et al., 2015; Nowacki et al., 2015; Meningher et al., 2017; Mu et al., 2020) and *F. gigantica* (Guo and Guo, 2019). These findings have stimulated interest in the possible value of worm-derived miRNAs as diagnostic biomarkers for helminthiases [reviewed by Mu et al., (2021)].

Alongside this diagnostic interest, recent efforts have focused on investigating the immunomodulatory potential of secreted helminth miRNAs (Arora et al., 2017). For example, *H. polygyrus* EV miRNAs can be internalized by the mouse epithelial cell line Mode K, and inhibit host type 2 innate immunity (Buck et al., 2014). In line with this, miR-10 contained in *Schistosoma mansoni* EVs, internalized by T helper cells, inhibits NF-κB activity essential for Th2 differentiation (Meningher et al., 2020). Several miRNAs of *S. japonicum* were reported to be involved in the regulation of host macrophages by inducing TNF-α production and monocyte proliferation (Liu et al., 2019). In another case, a particular *S. japonicum* miRNA (miR-3096) showed antitumor effects both *in vivo* and *in vitro* (Lin et al., 2019).

Numerous miRNAs have been identified within the EVs secreted by *F. hepatica* (Fromm et al., 2015). Moreover, *F. hepatica* EVs can be internalized by host macrophages (de la Torre-Escudero et al., 2019) and their miRNAs have been detected in macrophages of infected animals (Tran et al., 2021), indicating that *F. hepatica* secreted miRNAs, either in vesicles or free, could also be regulating host genes. However, miRNAs are not the only small RNA class with potential regulatory roles detected in the EVs of helminths (Cucher et al., 2021). To further characterize possible liver fluke regulatory RNAs, we compiled and analyzed the repertoire and expression of diverse small non-coding RNAs (sncRNA) in the key stages in the invasion and establishment of *F. hepatica* infection in the definitive host. Additionally, we studied the profile of sncRNAs contained in EVs released by the adult stage. Diverse miRNAs show expression differences across the life stages analyzed, and by miRNA target prediction both on the parasite and hosts genes, we attempted to gain insights in the regulation of the internal homeostasis of the parasite and its interaction with the host. Beside these, we found a restricted set of tRNA fragments (tRFs) that are abundant in all samples analyzed. Interestingly, those enriched in EVs can form homodimer structures that are, potentially, more stable and resistant to degradation by nucleases. Furthermore, we identified a new class of ncRNA previously unreported in trematodes, vault RNAs (vtRNAs), that are abundant in EVs.

## Methods

### Data Generation and Sequencing

Analyzed samples corresponding to diverse life stages (including dormant (MD) and *in vitro* activated metacercariae (MA), and adult worms (AD1; AD2) were generated by us and sequenced at The Genome Center at Washington University in St. Louis, Missouri, USA (WUGSC) within the frame of *F. hepatica* genome project (study SRP040521), (McNulty et al., 2017). Briefly, total RNA was extracted using mirVana Total Isolation kit (Thermo Fisher) in large (>200 bp) and small (<200 bp) fractions. Small RNA sequencing libraries were constructed using the NEBNext Multiplex Small RNA Library Prep Set for Illumina (New England Biolabs, Beverly, MA) according to the manufacturer’s instructions, and sequenced on the Illumina platform. Sequencing data is publicly available under accession numbers: SRR3584125 [MD], SRR3584124 [MA], SRR3584126 [AD1], SRR3584122 [AD2]. Newly excysted juveniles (NEJ [SRS862512]) data have been generated and analyzed by us previously (Fontenla et al., 2015).

EV samples were obtained from 50 adult worms recovered from a naturally-infected cow at a local abattoir in Dungannon, Northern Ireland. Briefly the flukes were thoroughly washed with PBS (3 x 200ml) to void their gut contents and then maintained in RPMI 1640 culture medium containing 0.1% glucose, 100 U penicillin and 100 mg/ml streptomycin (Sigma), at 2 worms/ml for 5 h at 37°C. EVs were isolated from the culture media by differential centrifugation as described by Cwiklinski et al. (2015) and treated with RNAase (Qiagen) to remove extracellular RNA not packaged within the EV. Total RNA was isolated from EV pellets using TRizol (Life Technologies) according to the manufacturer’s instructions. Small RNAs were subsequently isolated from the *F. hepatica* EV RNA preparations using the SeraMir kit (SystemBio). Libraries were generated with the TailorMix Micro RNA Sample Preparation version 2 protocol (SeqMatic LLC, Fremont, USA), amplified and Illumina sequenced. EV library construction and next generation sequencing was performed by System Biosciences, US. Sequencing data was deposited at the SRA repository with the accession number PRJNA782636.

All the data are freely available from SRA repository (https://www.ncbi.nlm.nih.gov/sra) with the given accession numbers. Quality and consistency of samples were evaluated by multidimensional scaling (MDS) (Mead, 1992) showing that they were comparable, despite being obtained from field isolates since lab pure lines or strains are still unavailable for *F. hepatica.*

### Quality Control and Genome Mapping

Trim galore (https://www.bioinformatics.babraham.ac.uk/projects/trim_galore/) was used to trim adapters and to remove reads shorter than 18 bp or with a phred score lower than 20. The remining reads were mapped to PRJEB25283 *F. hepatica* genome (https://parasite.wormbase.org) with Bowtie (Langmead et al., 2009), allowing a maximum of 2 mismatches (option -v 2). The coordinates corresponding to mRNAs, tRNAs, rRNAs, snRNAs and repetitive regions of the genome (i.e. transposons, low complexity regions or tandem repeats) were parsed out from the genome annotation, and the sequences obtained with bedtools getfasta with option -s (Quinlan and Hall, 2010). Next, Bowtie was used to classify reads by subsequently mapping to each dataset. Remaining reads were used to detect novel and conserved miRNAs.

Additionally, the presence of host-derived sequences was assessed by re-mapping the reads that failed to map to the genome of *F. hepatica*, to the genome of *Bos taurus* (downloaded from https://www.ensembl.org/Bos_taurus/Info/Index) with Bowtie (option -v 2) (**Table S1**).

A pipeline with the commands and scripts used in the analysis is available at GitHub repository (https://github.com/santifo/miRNA_analysis/blob/main/Pipeline).

Since MDS did not show significant differences in small RNA species and quantities between duplicated samples, and also between dormant and activated metacercariae (**Figure S1**), samples were considered as duplicates, pooled and averaged by stage: metacercariae (MC), adult (AD) and extracellular vesicles (EV), for further analysis.

### Classification and Identification of Known and Novel miRNAs

Reads not mapped to functional RNAs were collapsed and remapped to the genome with Bowtie with identical options as before. SAM output files were converted to arf format with bwa_sam_converter.pl. MiRDeep2 pipeline (Friedländer et al., 2012). was used to identify and quantify conserved and novel miRNAs, with a “known” dataset consisting of miRNAs deposited in miRBase v22.1 (Kozomara and Griffiths-Jones, 2014) and other *F. hepatica* miRNAs identified previously, but not deposited in miRBase (Ricafrente et al., 2021). Putative novel miRNAs were manually inspected, removing those with a score ≤ 5 and/or that showed drifting in the read stacking and/or had poor folding. Furthermore, BLAST and SSEARCH tools available at miRBase were used to confirm that novel miRNAs were not detected previously in other species. Additionally, MAFFT with local alignment parameters (Katoh and Standley, 2013), was used to align novel *F. hepatica* miRNAs to all Lophotrochozoan miRNAs deposited in miRBase, to search for identical ‘seed’ homology. Furthermore, a BLASTn (e-value 1e-4) was implemented to inspect if the novel precursors detected in *F. hepatica* could be conserved in any of the other platyhelminthes genomes deposited in WormBase Parasite (https://parasite.wormbase.org). No homologies were found except in the sister species *F. gigantica* (**Table S2**).

### Statistical Analysis of miRNA Expression

Statistical analysis of miRNA expression was performed with DEGUST v4.1.1 (https://degust.erc.monash.edu/). Samples were grouped by stage; and analyzed with Voom/Limma method, with count per million (CPM) normalization. Differentially expressed miRNAs were defined from pairwise comparisons of the log_2_-transformed expression estimates, establishing a minimum fold change of 2 and a false discovery rate (FDR) corrected P-values lower or equal to 0.01. MiRNAs with a total count below 10 reads were removed from the analysis. A hierarchical clustering with hclust function, was applied to differentially expressed miRNA between metacercariae, NEJ and adult.

### MiRNA Target Prediction and Functional Enrichment of *F. hepatica* Gene Targets

*F. hepatica* conserved and novel miRNAs were used to predict targets in an iso-seq curated database of *F. hepatica* genes (FHISCDB). This database consisted of 7626 3’ untranslated regions (3’UTRs) annotated in PRJEB25283 assembly, and 1612 3’UTRs of novel transcripts obtained from a prediction based on remapping all RNA-seq data available in repositories, and iso-seq sequencing reads from intra snail stages of *F. hepatica* (Langleib et al. in preparation). Transdecoder (available at https://github.com/TransDecoder/TransDecoder/) was used to predict the open reading frame (ORFs) and 3’UTR on novel transcripts.

Three prediction algorithms were used: miRanda (Betel et al., 2010), PITA (Kertesz et al., 2007) and TargetScan v7.0 (Agarwal et al., 2015), with default options. Given that each algorithm implements different mechanisms to predict target sites [reviewed by (Peterson et al., 2014)], they usually differ in a few bases since they include different lengths outside the seed region. Therefore, to provide a consistent matching position of each target, we have reported the position on the 3’UTR matching the first base (start) of the “seed region” of the miRNA in column D of **Table S4**.

Genes defined in the FHISCDB were functionally reannotated with eggNOG-mapper (Huerta-Cepas et al., 2019). TopGO (Alexa and Rahnenfuhrer, 2019) (with option: statistic = “fisher”) was used to identify enriched gene ontology (GO) terms between the gene targets of differentially expressed miRNAs, GO terms with a P-values lower or equal to 0.01 were considered significantly enriched.

### miRNA Target Prediction in Mammals and Identification of Regulated Functions

The conserved and novel miRNAs of *F. hepatica* were used to predict complementary target sites in host species genes. 3’UTRs from sheep, cattle and human were downloaded from TargetScan database (http://www.targetscan.org/cgi-bin/targetscan/data_download.vert72.cgi). Although the initial idea was to use a 3 by 3 strategy, retaining only the targeted positions within the 3’UTR predicted by the three algorithms (miRanda, PITA and TargetScan v7.0) conserved in the three host species, the low quality of 3’ annotation in the sheep and cattle datasets resulted in many sites conserved only in human and one of the ruminant species. Consequently, we decided to retain all those targets identified by the three algorithms in the human dataset. As described previously, predictions were run with defaults options. DAVID database (Huang et al., 2009) was used to identify regulated functions in targeted genes, considering only the pathways regulated by two or more miRNAs. Furthermore, InnateDB (Breuer et al., 2013) was used to identify the genes related to immunity under regulation.

### Quantification and Folding of tRNA Derived Fragments

The reads that mapped to the tRNA database were quantified and classified using in-house scripts. Reads containing a terminal CCA-3’ anchor were subsequently quantified using a similar processing, except that the tRNAs of reference were modified by adding a ‘CCA’ trinucleotide anchor to their 3’ end. The output files were analyzed to obtain the information on alignment positions from first to end base found in the tRNA precursor. These positions were used to categorize the tRNAs fragments. The fragments resulting from the cleavage at the anticodon loop were named 5’ tRNA half fragment (5’tHF) and 3’ tRNA half fragment (3’tHF); if the fragment was contained but shorter than the 5’ or 3’ halves they were named 5’ fragments (5’tRF) and 3’ fragments (3’tRFs). The fragments that started at the 5’ or 3’ ends and were longer than the 5’tHF or 3’tHF were classified as 5’ tRNA long fragments (5’tLF) or 3’ tRNA long fragments (3’tLF). The fragments that were not contained within these categories or that corresponded to full tRNAs were poorly represented and were not further classified. For each tRNA fragment, the sequence with the highest count was used as input for *in-silico*, folding and dimerization prediction using the ‘Fold RNA Biomolecular’ tool of RNAstructure6.3 package (Bellaousov et al., 2013).

### Detection of Vault RNAs

Vault RNAs are short (80 to 150 nt) polymerase III transcripts, that show no sequence conservation except in two short regions, box A and box B, that correspond to internal polymerase III promoter elements. Since finding them by traditional homology methods is unpractical, we followed a modified version of the approach described by Stadler et al. (Stadler et al., 2009). We used the most conserved region, the box A, plus the 3’ flanking ‘TTACTTCG’ of *L. gigantea* and *H. robusta* obtained from Rfam database (RF00006 (https://rfam.xfam.org/).) to perform a relaxed BLASTn search in the genome of *F. hepatica*. Typical polymerase III-terminator motifs (consisting mainly of a run of T’s) were sought in the 150 pb region downstream of the blast HSPs hits.

Putative vtRNAs were further inspected with MEME suite (Bailey et al., 2009) to confirm the conservation of relevant motifs, and RNAfold (Lorenz et al., 2011) to evaluate the presence of the characteristic panhandle-like secondary structure. Next, we used fragrep2 (Mosig et al., 2006) pipeline to align and generate a *F. hepatica* specific pattern of vtRNAs with the conserved motifs. Fragrep2 tool implements an algorithm for detecting the pattern fragments that occur in a given order but are interrupted by non-conserved sequences of highly variable length. The pattern was used to re-search the genome. Additional putative vtRNAs detected were also evaluated as described. The genomic locations of all vtRNAs were inspected to confirm that no other features were annotated and that the vtRNAs genes were being expressed.

## Results

### Small Non-Coding RNAs Are Present in All Stages, and Also in Secreted Vesicles

Small RNAs produced by metacercariae, newly excysted juveniles (NEJ) and adults of *F. hepatica* were analyzed and compared with novel data generated from the sequencing of small RNAs present in extracellular vesicles (EV) released by the adult stage.

**Figure 1:**
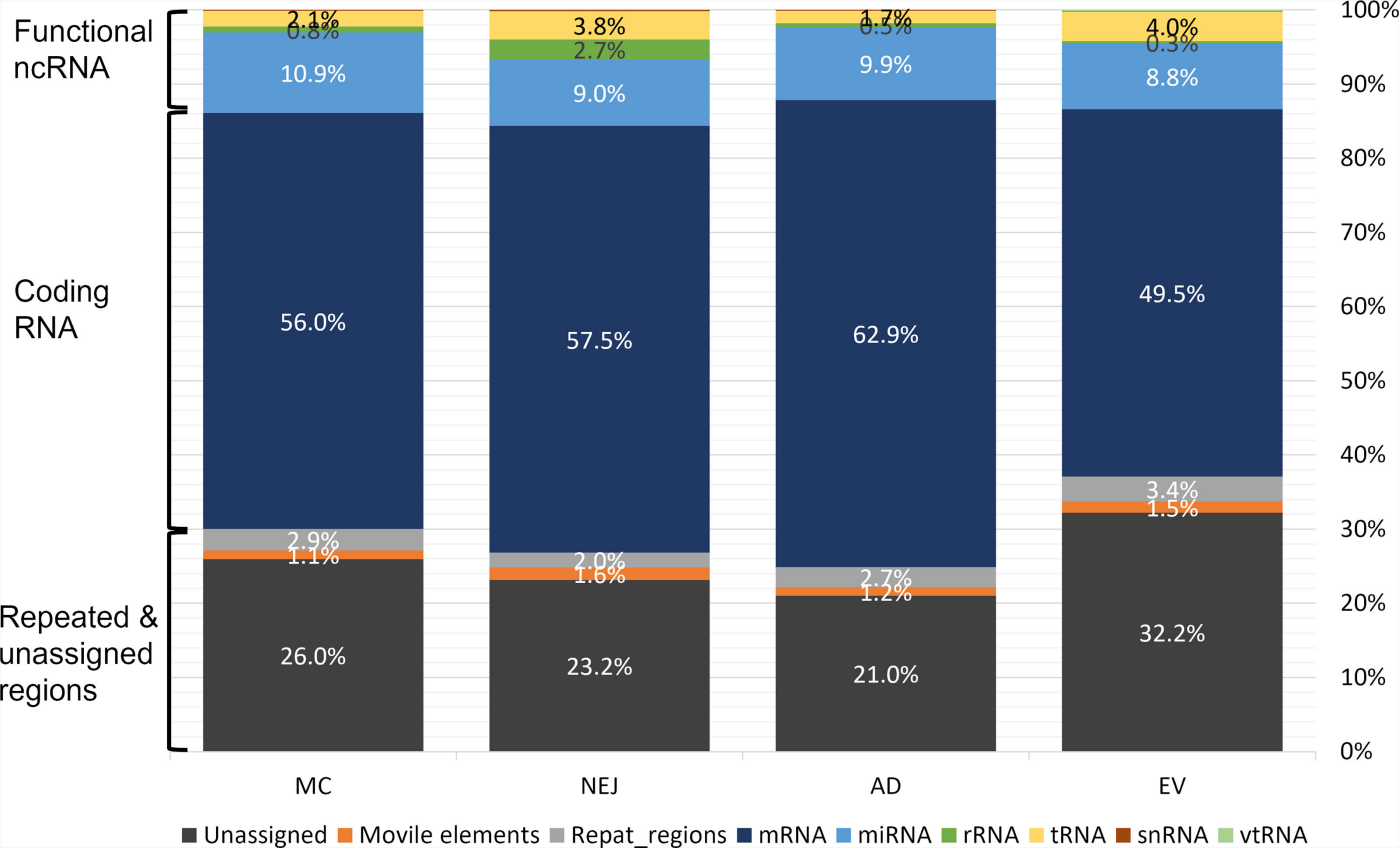
Mapping of small RNA sequences from different stages of *Fasciola hepatica* to the genome. Samples were obtained from metacercaria (MC), newly excysted juvenile (NEJ) adult worms (AD), and adult-released extracellular vesicles (EVs). Mapping reads were classified in three main categories: functional non-coding (nc)RNA (rRNA, tRNA, small nuclear (sn)RNA, vault (vt)RNA and miRNA), coding RNAs (mRNA) and those associated to repeat and unassigned regions (mobile elements (e.g., transposons), regions with repeated sequences or regions that remain unannotated).

Sequencing data samples were analyzed in parallel as described in the methods section. Despite differences in the initial number of raw reads, roughly 77% passed the quality filtering and trimming stages (**Table S1**). More than 80% of these high-quality reads mapped to the genome of *F. hepatica* (PRJEB25283), except for NEJ where the mapping values were lower at 66% (**Table S1**). The length distribution of the reads that mapped to the genome showed a prominent peak at 21-22 nt in all samples, consistent with the presence of miRNAs, and a secondary less prominent peak at 31-33 nt that is consistent with our previous analysis (Fontenla et al., 2015) and that corresponded to sequences that mapped to tRNAs (see below) (**Figure S2**). Reads that mapped to the genome were subsequently classified by mapping to different datasets (see Methods). While the majority of the reads mapped to coding RNA and repeated regions, a small but significant fraction corresponded to putative functional non-coding RNA. On average, 13.7% of the reads corresponded to the category of functional ncRNAs that grouped: miRNAs, tRNAs, rRNAs and vtRNAs (described here for the first time in trematodes). In all samples, miRNAs were the most abundant ncRNA followed by tRNA-derived fragments (**Figure 1**).

### MiRNA Profiles Vary Greatly Between Different Stages

**Figure 2:**
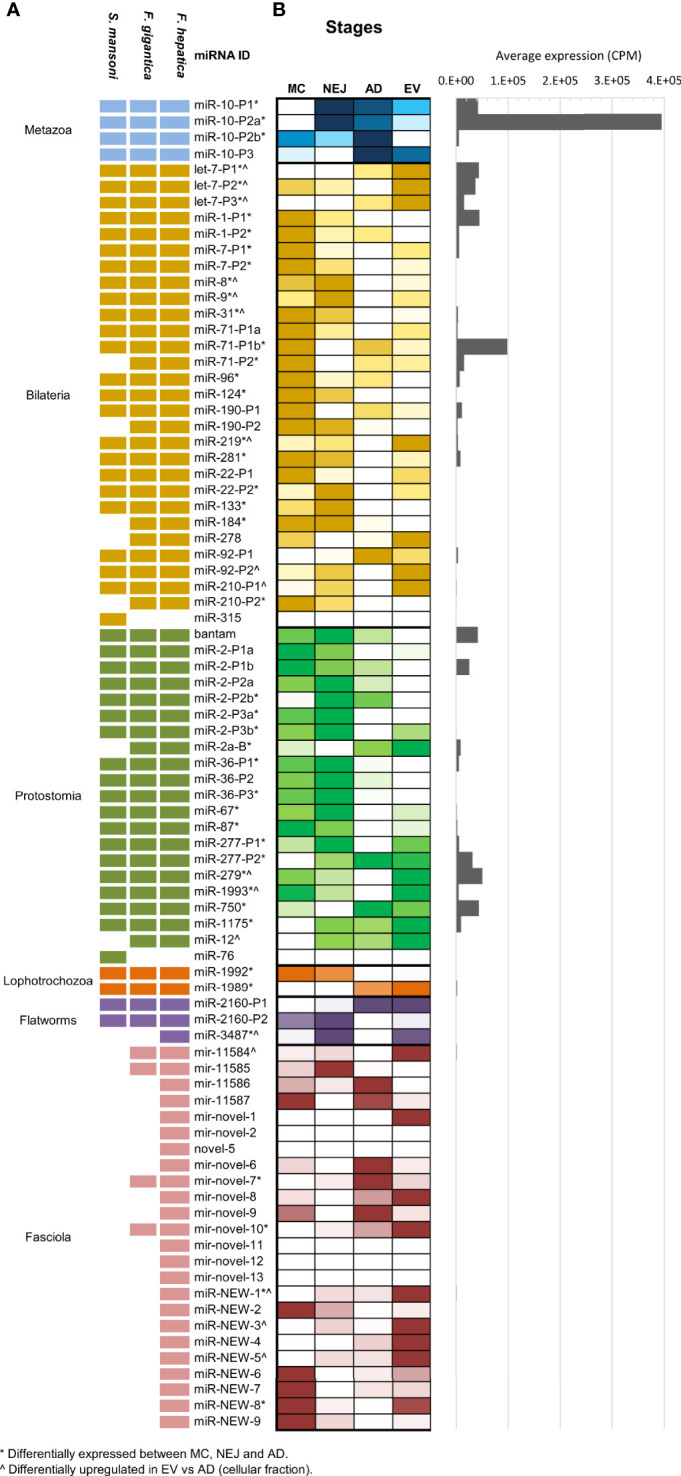
MiRNA complement and relative abundance across species and stages. **(A)** Updated miRNA complements in *F*. *hepatica*, *F*. *gigantica* and *S. mansoni*. individual miRNAs belonging to the same miRNA family have the same id plus an individual precursor number. Shaded blocks indicate evolutive conservation across: Metazoan (light blue), Bilateria (yellow), Protostomia (green), Lophotrochozoa (orange), flatworms (purple) or Fasciola species (pink). Empty (white) blocks indicate that the miRNA has not been detected in the species **(B)** Heatmap depicting the comparative abundance of individual miRNAs between MC, NEJ, AD stages, and the adult EV fraction. Read counts were normalized to count per million (CPM) and color scale was applied. White shaded blocks indicate lower expression, darker colors indicate high abundance. (*) Indicates significant variations between cellular fractions of MC, NEJ, and AD (FDR ≤ 0.01 & Log2FC ≥ 2). (^) Indicates significantly upregulated in the EV fraction respect to the somatic fraction of AD (FDR ≤ 0.01 & Log2FC ≥ 2). A histogram with the average expression of each miRNA shows that only a reduced set are always upregulated.

Compiling all the data published so far on *Fasciola hepatica* miRNAs (**Table S2**), we observe that *F. hepatica*’s miRNome is still reduced with respect to other Lophotrochozoans or even free-living flatworms [flatworms are expected to share at least 46 miRNA families (Tarver et al., 2013)]. As described previously by us and others (Fromm et al., 2013; Fontenla et al., 2015), neodermatans are characterized by a reduction in the conserved miRNA families. At present the miRNome of *F. hepatica* is composed of 34 conserved families (13 of them with more than one miRNA within the family). An almost identical complement of miRNAs was recently reported in *F. gigantica* (Hu et al., 2021) and previously in *S. mansoni* (Ovchinnikov et al., 2020) (summarized in **Figure 2A**), indicating that these losses could have been in the origin of the adaptation to parasitism of the clade. In addition, to the conserved miRNA, 15 novel *F. hepatica* exclusive miRNAs have been described previously (Ricafrente et al., 2021), four of which were also confirmed in the miRNA complement of *F. gigantica* (Hu et al., 2021). Here, we further extended the Fasciola specific miRNome by adding nine new well supported miRNAs (fhe-miR-NEW-1 to 9 in **Figure 2** and **Figure S3**), with seven of them, also predicted to be conserved in the *F. gigantica* genome (**Table S2**).

When the normalized expression of miRNAs was compared across the intra-mammalian stages, we observed that a reduced set of evolutionary conserved families are very predominant (**Figure 2B** and **Table S3**). The metazoan conserved fhe-miR-10-P2a (named fhe-miR-125b in miRBase) was the most highly expressed miRNA across all stages and in the EV fraction of the adult stage, despite variations in the proportional representation. Other conserved miRNAs highly expressed across stages and EVs were fhe-miR-71-P1b found in all bilaterians, and bantam, a miRNA characteristic of protostomes (**Figure S4**).

Interestingly, 13 of the 28 miRNAs conserved between Bilaterians were significantly more expressed in MC respect to the other stages [(*) in **Figure 2B**], while more than a third of the protostomian specific miRNAs showed higher abundance in NEJ (**Figure 2B**). miRNAs conserved across Lophotrochozoans and Flatworms showed low expression in all samples, despite significant variations for fhe-miR-1992, fhe-miR-1989 and fhe-miR-3479. However, when we compared our results with those described in the sister species *F. gigantica* (Hu et al., 2021), we noticed that several lophotrochozoan and flatworm conserved miRNAs (e.g. miR-1992, miR-1989, miR-2160-P1 & -P2, and miR-11584), showed higher expression in egg and the intra-snail stages (**Figure S5**). Significant variations were also detected for several *Fasciola* specific miRNAs across diverse stages.

### MiRNAs as Regulators of Development and Metabolism in *F. hepatica*

**Figure 3:**
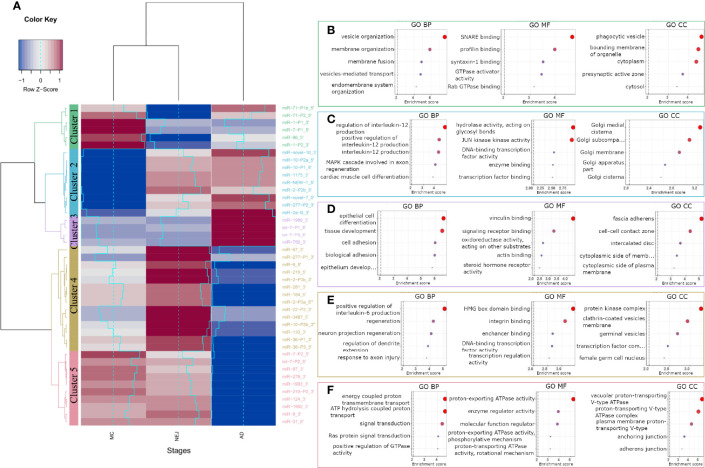
GO terms enrichment of differentially expressed miRNAs between metacercariae, NEJ and adult stages. **(A)** Heatmap of the differences in the expression between stages. MiRNAs were grouped into 5 clusters based on their expression profiles: cluster 1 (green), cluster 2 (blue), cluster 3 (purple), cluster 4 (yellow) and cluster 5 (pink). A gradual change in color ranging from blue to red indicates the low-to-high expression, additionally light-blue line reflects the variation in the Log2FC. **(B–F)** Top 5 biological process (BP), molecular function (MF) and cellular component (CC) terms ordered by p-value, enriched in the predicted targeted genes of clusters 1-5, respectively (a color code like in A was applied). Enrichment score was calculated as -Log of p-value, terms with a score ≥ 2 (p-value = 0.01, dashed line) were considered significant.

To analyze the role of miRNAs in the regulation of the homeostasis of *F. hepatica*, we classified the differentially expressed mature and two co-mature star miRNAs into five expression clusters (**Figure 3A**). Target sites in the transcripts of *F. hepatica* were predicted for the five clusters (**Table S4**). We found 8851 targeted positions in 3369 transcripts predicted by the three algorithms used, including 423 transcripts corresponding to novel genes predicted by us (see *Methods* section, Langleib et al. in preparation). GO enrichment analysis of the targeted genes by each cluster of miRNA suggests that they might be regulating functions related to development, signaling pathways and transport, among others (**Figures 3B–F** and **Figures S6A–E**).

Cluster 1 represents a set of miRNAs highly expressed in MC while lowly expressed in NEJs, probably highlighting the regulation of relevant genes in the first interaction with the host and in the invasion process. Interestingly the genes targeted by cluster 1 miRNAs are enriched in function and processes associated with vesicle organization and transport, and membrane fusion (**Figure 3B** and **Figure S6A**).

MiRNAs strongly downregulated in MC while upregulated in NEJ and adults are grouped in cluster 2 (**Figure 3A**). Genes related to the Golgi are enriched within cluster 2 targets, suggestive of a role in vesicular cargo maturation and trafficking. Since some of the enriched targets to Cluster 2 miRNAs are genes involved in positive regulation of IL-12 signaling, the upregulation of these miRNAs in NEJ and adults might have a role dealing with the IL-12 mediated responses generated by the host macrophages and dendritic cells (**Figure 3C**). Other functions highlighted in the top 20 processes enriched like “response to inorganic substances” and “response to temperature stimuli” (**Figure S6B**) could be related to sensing stimuli relevant for excystment and development.

Cluster 3 includes miRNAs highly expressed in adults, and enrichment terms on targeted genes include: cell differentiation and tissue morphogenesis of epithelial cells and cell to cell contact. These processes are very relevant in the maturation of juvenile forms, during the formation of the syncytial tegument (**Figure 3D** and **Figure S6C**).

On the other hand, Cluster 4 contains miRNAs highly expressed in NEJ but strongly downregulated in adults. Here we found functions associated with nervous development, transcription regulation and interestingly some terms related to germinal functions, that might be associated with the production of eggs in the adult stage (**Figure 3E** and **Figure S6D**).

Cluster 5 represents miRNAs strongly downregulated in adults but expressed in MC and NEJ, and here enriched terms are related to signal transduction particularly those associated with vacuolar proton transport ATPases. These enzymes are associated with the acidification of vacuoles, a requisite for the function of several proteases relevant in feeding and the interaction with the host (**Figure 3F** and **Figure S6E**).

### A Reduced Set of miRNAs Are Highly Represented in the EVs of Trematodes

The analysis of miRNAs cargo in EVs showed a sharply reduced set of miRNAs with a highly biased enrichment of particular miRNA signatures, e.g., the top 10 abundant miRNAs corresponding to 87.9% of the miRNA population. Additionally, 16 miRNAs were significantly upregulated in EVs with respect to the adult stage (**Figure 2B**), most prominently fhe-let-7-P1, -P2, -P3 and fhe-miR-279 that were among the most highly expressed miRNAs in the EV data but were significantly less dominant in the cellular fraction of the adult stage (**Figure S4**).

**Figure 4:**
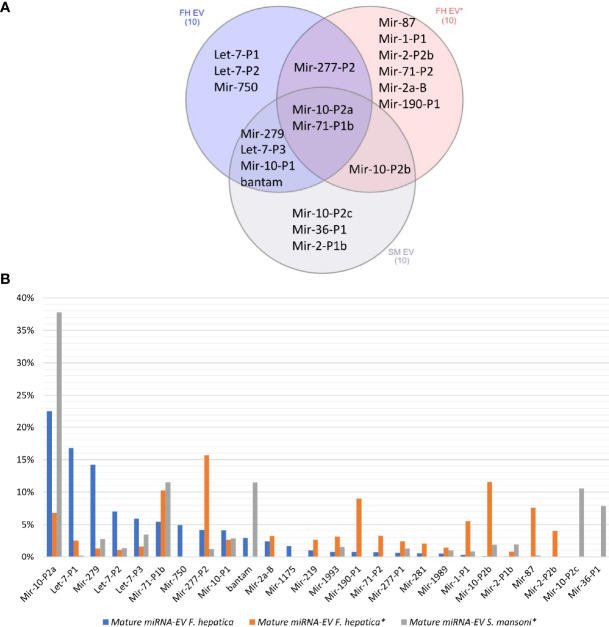
**(A)** Venn diagram of the top 10 abundant miRNAs in the EVs of two *F. hepatica* experiments and *S. mansoni*. **(B)** Bar plot shows the percentage of the total represented by the most abundant orthologous miRNAs. (*) Data generated by (Ovchinnikov et al., 2020).

When we compared our *F. hepatica* EV miRNA profile with those previously reported for *F. hepatica* and *S. mansoni* (Ovchinnikov et al., 2020), we observed that the same reduced set of miRNA families were overrepresented but with differences in ranking between species and studies (**Figure 4**). For example, miR-10-P2a and miR-71-P1b were in the top 10 of the three experiments (**Figure 4A**), with the former being the most abundant both in the cellular and EV fractions in our samples of *F. hepatica*’s and in the *S. mansoni* study (Ovchinnikov et al., 2020) (**Figure 4B**). Interestingly, we observed more similarities between the profiles we obtained and those reported for *S. mansoni* than with the other *F. hepatica* experiment (**Figure 4A**). If we extend the analysis to all the adult EV miRNAs detected in trematodes (reviewed by Sotillo et al., 2020; Avni and Avni, 2021), we consistently observed that the top 10 abundant miRNAs accounted for more than 80% of the total miRNA population. These highly expressed miRNAs correspond to ancient metazoan miRNA families, including miR-10, let-7, miR-71 and the lophotrochozoan specific families: bantam, miR-2, miR-279 and miR-277, among others (**Table S5**).

### Analysis of Putative Host Genes Targeted by Parasite EV-Derived miRNAs Highlights Relevant Regulatory Functions

**Figure 5:**
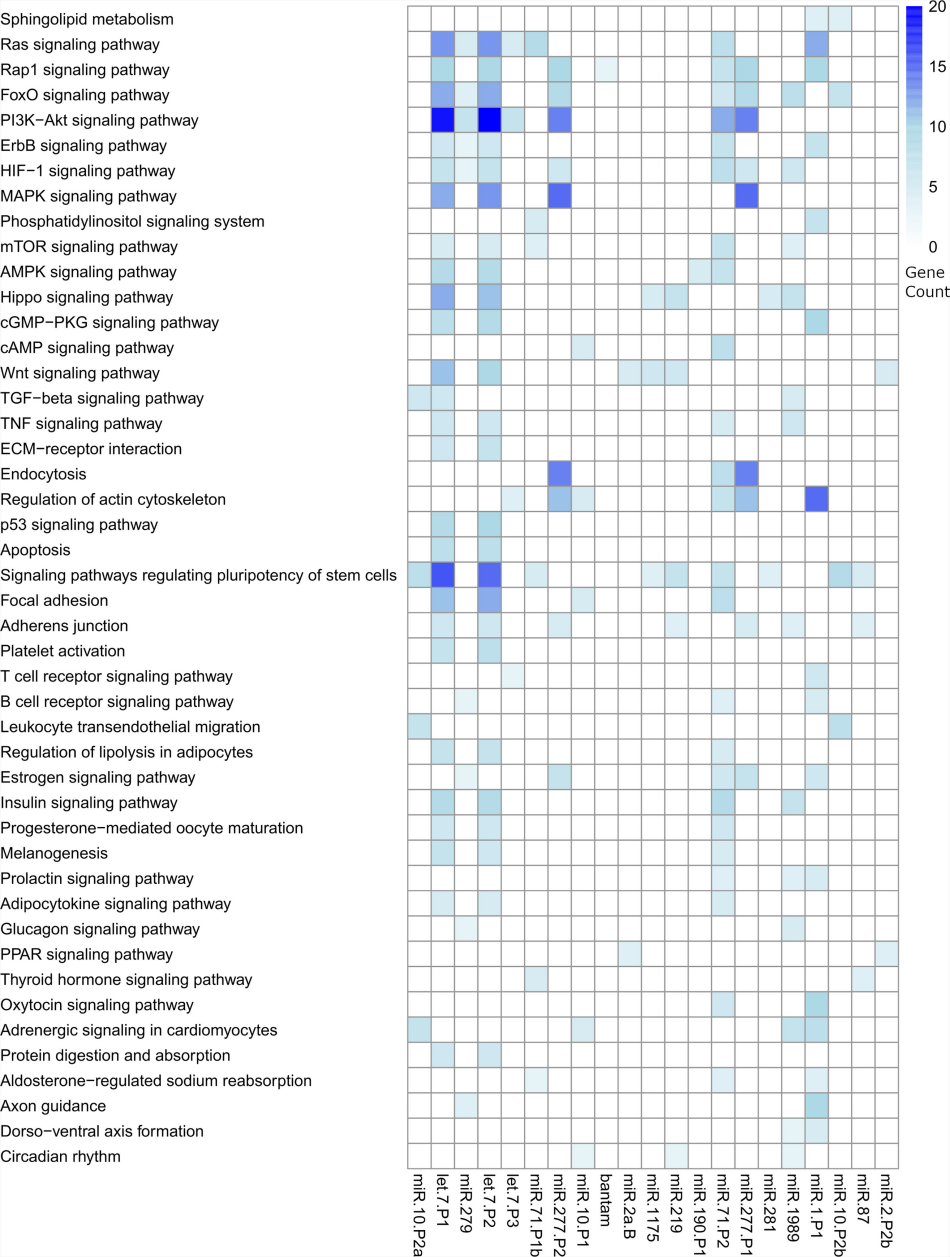
Heatmap showing the significant pathways regulated by the miRNAs contained in the EVs of *F. hepatica*. DAVID database was used to calculate enriched pathways. Counts of genes targeted within each pathway are indicated by a color scale. The list of targeted transcripts is reported in **Table S6** and those related to immunity are presented in **Table S7**.

We predicted complementary binding position in the 3’ UTR of host genes for the most abundant *F. hepatica* miRNAs detected in both EVs studies (indicated in **Figure 4B**). We found 7105 targeted sites, with identical coordinates between the algorithms used, in 3385 different genes (**Table S6**). Next, we used DAVID database (https://david.ncifcrf.gov/tools.jsp) to identify enriched pathways only retaining those that were regulated by more than one miRNA. Several fundamental signaling pathways such as Ras, MAPK, PI3K-Akt, ErgB and Wnt emerged as putative enriched targets (**Figure 5**). Additionally, pathways specifically related to immune response such as TNF signaling, Platelet activation, T cell receptor signaling, B cell receptor signaling and leukocyte transendothelial migration were also found under regulation. Therefore, we consulted InnateDB (https://www.innatedb.com/), to better identify the genes related to immunity under regulation by the miRNAs contained in the EVs of *F. hepatica*. From the initial gene set identified, we only retained 77 genes that were targeted in two or more positions, as they may be under a more rigorous regulation by the miRNAs of *F. hepatica* (**Table S7**). Interestingly, the miRNAs with most targets among these genes (fhe-let-7-P1 & -P2, fhe-miR-10-P2a & -P2b, fhe-miR-1 and fhe-miR-277-P1 & -P2) were also among the most abundant miRNAs both in our data and in the previous *F. hepatica* EVs study (Ovchinnikov et al., 2020).

### TRNAs-Derived Fragments Were Detected in EVs and in All Somatic Samples

**Figure 6:**
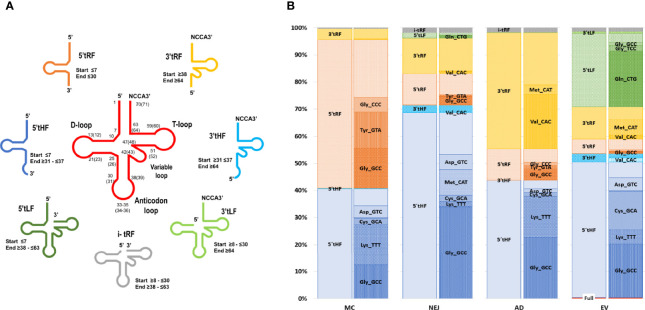
Expression profiles across samples and folding of tRNAs-derived fragments. **(A)** Depiction of tRNA fragments detected in the study, a color code was used for each class. **(B)** Bar charts grouped by sample showing the percentage of each tRNA fragment class detected and proportion of individual fragments related to the tRNA of origin and the sequence of the anticodon.

Similar to what we have previously reported in NEJ only (Fontenla et al., 2015), reads mapping to tRNAs corresponded to a longer class of small RNA sequences (31-35 nucleotides in length, with a prominent peak at nucleotide 32) (**Figure S7**). These reads corresponded predominantly to diverse fragments generated from cleavage of a restricted set of tRNAs. By inspecting read alignments, we detected diverse cleavage points over the mature tRNAs resulting in distinct type of fragments (classified and described in Methods, **Figure 6A**). Overall, the most abundant class across all samples were the 5’halves of tRNAs (5’tHF) generated by cleavage in the anticodon loop, but also other fragments were detected. Shorter 5’ tRF were frequently detected in metacercarial samples and were the product of cleavage before the anticodon loop or in the D loop of the precursor tRNA, while short 3’fragments were abundant in adult samples and were produced by cleavage at the T loop. Longer 5’ fragments (5’tLF) generated by cleavage at the T loop were observed in EVs (**Figure 6B** and **Figure S7**). Full length tRNAs were only found in EVs, representing less than 5% of the tRNA mapping reads, although 65% of them correspond to the Selenocysteine tRNA^Sec_TCA^ (**Table S8**).

Similarly, a marked skew in representation of tRNA precursors was observed in all fragment classes. Within 5’tHFs, five precursors corresponding to tRNA^Gly_GCC^, tRNA^Lys_TTT^, tRNA^Cys_GCA^, tRNA^Met_CAT^ and tRNA^Asp_GTC^ were preferentially retained in all the samples, although in different proportions (**Figure 6B**). 5’tRFs were particularly abundant in the MC stage, with tRNA^Gly_GCC^, tRNA^Tyr_GTA^, tRNA^Gly_CCC^ representing more than half of them. These same 5’tRF species were also enriched in other stages and EVs although they were proportionally less significant. In adult samples, 3’tRF were abundant, enriched particularly in tRNA^Val_CAC^, generated by cleavage at the T-loop (in 99.4% of the cases), and to a lesser extent in the 3’tRF derived from tRNA^Met_CAT^. Within EVs, a substantial fraction (26%) of tRNA derived molecules were 5’tLFs, (corresponding to the prominent peak at nucleotide 53 in **Figure S7**), but again with a very skewed representation, with tRNA^Gln_CTG^ derived molecules representing more than 75% of their class.

**Figure 7:**
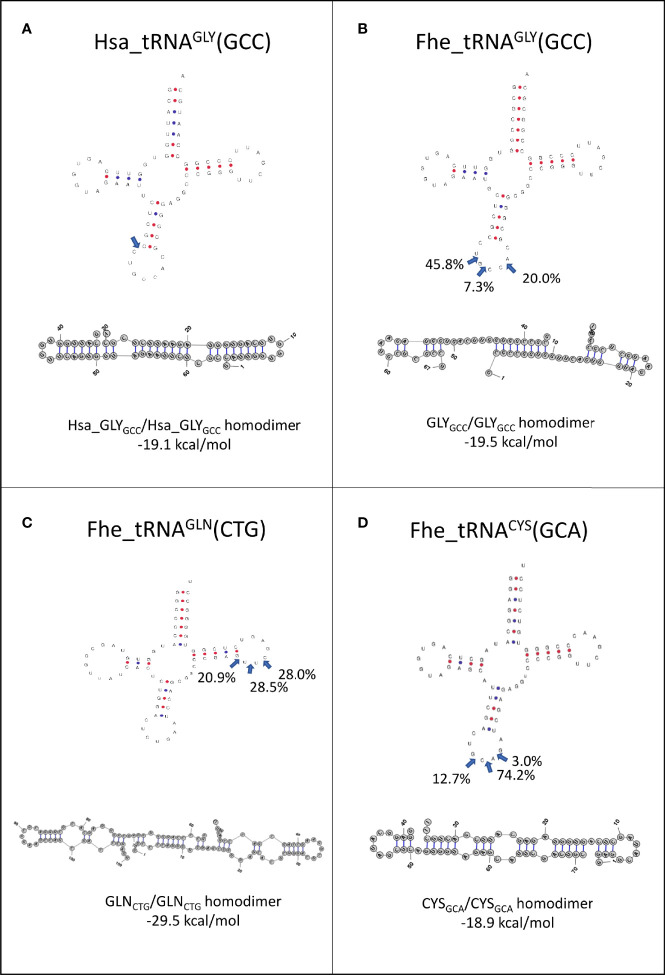
Folding of tRNAs-derived fragments. Full length tRNA secondary structures were predicted with tRNAscan-SE (http://lowelab.ucsc.edu/tRNAscan-SE/), position and frequency of the most frequent cleavage sites in EVs are indicated (blue arrows). Homodimer structures and energy in kcal/mol for: **(A)** Hsa_Gly_GCC_, **(B)** Fhe_Gly_GCC_, **(C)** Fhe_Gln_CTG_ and **(D)** Fhe_Cys_GCA_ as predicted by RNAstructure6.3 for the most abundant fragment.

Since it has been proposed that homo and heterodimerization can stabilize tRNA fragments, preventing their degradation in the extracellular medium (Tosar et al., 2018), we analyzed the preferred cleavage site of the tRNAs packed in EVs, and used the resulting fragments to predict if homodimer structures were possible (**Figure 7**). Interestingly, 5’tHF-tRNA^Gly_GCC^, 5’tLF-tRNA^Gln_CTG^ and 5’tHF-RNA^Cys_GCA^, the most abundant in EVs, formed homodimeric structures that hid the 3’ ends in a similar way to what was described for the 5’ tRNA^Gly_GCC^ tHFs of humans (see **Figures 7A–D**). Despite similarities, the *F. hepatica* Gly-GCC homodimer is maintained by an uninterrupted 8 nt-long stretch of Watson and Crick or G:U pairing, while there are 12 base pairings in the human counterpart (**Figures 7A, B**). Additionally, we observed the formation of a stable homodimer from a 5’tLF, generated by cleavage at the T-arm of tRNA^Gln_CTG^ (**Figure 7C**, blue arrows), around the pseudouridine, instead of the anticodon loop. This Gln_CTG_/Gln_CTG_ homodimer tRDFs is bonded by the interaction of 10 nt, with an uninterrupted stretch of 6 nt.

While the 5’tHF-RNA^Met_CAT^ generates homodimers with hidden 3’ ends, only 4 bp bonded the monomers, suggesting it might be more prone to dissociation (**Figure S8A**). In contrast, the homodimers predicted with other frequent tRNA fragments as 5’tHF-RNA^Lys_TTT^, 5’tHF-RNA^Asp_GTC^ or 5’tLF-RNA^Gly_TCC^ generated structures with free 3’ends that are probably more susceptible to degradation by exonucleases (**Figures S8B–D**). No stable heterodimers were predicted for any of the tRNA fragments analyzed.

### Vault (vt)RNAs Are Present in All Stages but Enriched in EVs

**Figure 8:**
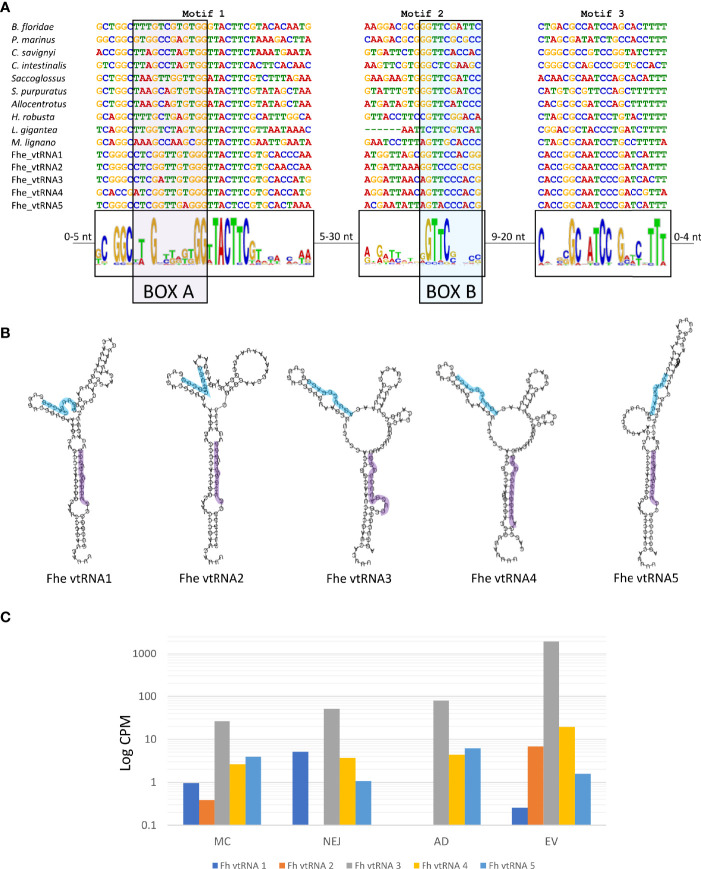
Structure and expression of vtRNAs detected in the genome of *F. hepatica.* **(A)** *F. hepatica* vtRNAs were compared with vtRNAs detected in other species; chordata: *Petromyzon marinus*, *Branchiostoma floridae*, *Ciona intestinalis* and *C*. *savigni*; hemichordate: Saccoglosus; Echinodermata, *Strongilocentrotus purpuratus* and Allocentrotus; Annelida: *Helobdella robusta*; Mollusca: *Lottia gigantea*; Platyhelminthes: *Macrostomum lignano*. Motifs 1 and 3 were detected with MEME suite, motif 2 was inferred from the sequence alignment. Motif 1 and motif 2 contain the internal promoter sites of RNA polymerase III [indicated as BOX A (highlighted in purple) and BOX B (highlighted in light blue)]. The sequence range in the variable region between the motifs are indicated. **(B)** Fhe-vtRNAs panhandle-like secondary structure, Box A is highlighted. **(C)** Bar chart with the expression profile of fhe vtRNAs in the stages analyzed. Reads were averaged between samples, normalized to count per million and a logarithmic scale was applied to improve data visualization.

Besides the well-known presence of miRNA and tRNA fragments in EVs, the detection of the major vault proteins (MVP) as part of the EVs cargo (Cwiklinski et al., 2015) raised the question if the corresponding vtRNAs were also present. Vault particles are short polymerase III transcripts with lengths varying between 80 and 150 nucleotides, with sequence conservation restricted to two short regions, box A and box B, that correspond to internal polymerase III promoter elements (**Figure 8A**).

For the detection of vtRNAs, we applied a combination of relaxed homology and pattern searches, motif detection, structural prediction and manual curation (described in detail in ‘Methods’). We detected 5 putative vtRNAs that passed the filtration process. By using MEME suite, we found two common motifs between the putative *F. hepatica*’s vtRNAs and recognized animal vtRNAs sequences (motifs 1 and 3 in **Figure 8A**). While the conserved box A was included in motif 1, the box B included in motif 2 was not detected by MEME suite and was inferred from the sequence alignment. The sequence conservation of box B was very low being limited mostly to the sequence ‘GTTC’ or one nucleotide variations of it (**Figure 8A**). The folding prediction of the putative vtRNAs produced panhandle-like secondary structures with an extended stem-loop connecting 5’ and 3’ ends similar to those characterized in other animals (**Figure 8B**) (Stadler et al., 2009).

Interestingly four of the five putative vtRNAs detected were clustered in a 133.3 Kb region on the scaffold 1448, with the remaining structure (vtRNA4) in another scaffold (scaffold 1781, **Table S9**). While all of them were expressed in all stages analyzed, vtRNA3 was predominant in all samples, and particularly enriched in EVs (more than 20-fold in relation to the corresponding adult stage) (**Figure 8C**).

When we inspected the genomic regions predicted to contain vtRNA in the wormbase parasite database, we found good RNA-seq reads coverage in samples from different stages and diverse experiments, confirming their expression. Once again, vtRNA3 was the most expressed overall (**Figure S9**).

## Discussion

In this study we analyzed the presence of diverse small RNA populations across distinct life stages of *F. hepatica*, and those present in vesicles secreted by the adult stage. Although samples were obtained and sequenced at different times, those from similar stages tend to cluster in multidimensional analysis, suggesting they represent the population consistently found at these stages (**Figure S1**). Furthermore, read data were normalized to account for differences in sequencing depth. A complex set of different small RNAs was identified in all samples analyzed, composed by miRNAs, tRNA fragments and the longer vtRNAs.

### MiRNAs Are Associated With Host Invasion and Development of *F. hepatica*

Our analysis of the miRNome of the main stages of *F. hepatica* associated with the definitive host, adds nine novel miRNAs to those already described and recompiled recently (Ricafrente et al., 2021). The comparison with a recent study in the sister species *F. gigantica* (Hu et al., 2021), confirms the presence of 34 conserved families and a growing set of miRNAs described so far only in Fasciolidae.

Additionally, we *in-silico* correlated the differentially expressed miRNAs with the putative biological processes under regulation, with results suggestive of relevant roles in the normal development of the parasite and possible roles in the invasion process. Even though, our results await further experimental validation, the regulatory roles of miRNAs are well known (Bushati and Cohen, 2007). Studies in the model animals *C. elegans* and *Drosophila*, show that the most conserved animal miRNAs are abundant and usually involved in core developmental process, while scarce miRNAs are usually related to specific functions, and sometimes expressed in very restricted groups of cells or tissues (Alberti and Cochella, 2017). In this sense, in planarians, particular miRNA families (also conserved in *F. hepatica)*, were found to show preferential expression in neoblasts or to be upregulated in the regenerating tissue (González-Estévez et al., 2009; Sasidharan et al., 2013; Cao et al., 2020). It is possible that similar cell type-miRNA preference occurs in *F. hepatica*, however, additional experiments are needed to corroborate this hypothesis. In parasitic neodermatans, the role of miRNAs in the regulation of cellular processes, up until now, was mainly restricted to descriptions in Schistosomes (Sun et al., 2014; Zhu et al., 2016; Protasio et al., 2017; Yu et al., 2019), *Echinococcus* spp. (Guo et al., 2017; Macchiaroli et al., 2017; Pérez et al., 2019; Bai et al., 2020; Macchiaroli et al., 2021) and more recently *F. gigantica* (Hu et al., 2021). Orthologous miRNAs of those described in these studies are present in *F. hepatica*, suggestive of common regulatory pathways. Interestingly, our enrichment study highlighted different clusters of miRNA expression, that in turn might regulate diverse processes. Is interesting that several of the miRNAs (fhe-miR-71-P1b, fhe-miR-71-P2, fhe-miR-1-P1, fhe-miR-1-P2, fhe-miR-96 and fhe-miR-7-P1) might be regulating the formation and release of vesicles. A recent characterization of EVs in different developmental stages of *F. hepatica* has reported the secretion of large amounts of different types of vesicles in NEJ. These vesicles were found to be pre-formed in the metacercariae (Sánchez-López et al., 2020). Furthermore, secretory vesicles with distinct morphologies were observed in the tegument of *F. gigantica* NEJs before and after penetration of the host intestine indicating that secretion by this life-cycle stage requires a fine level of control (Hanna et al., 2019). Therefore, the change in the expression of the miRNAs of cluster 1 (**Figure 3A**) could be, at least in part, regulating the release of the preformed vesicles until appropriate signals from the host trigger their release from the apical plasma membrane.

### MiRNAs Contained in *F. hepatica* EVs May Target Immune-Related Host Genes

During the establishment of the infection the strategy adopted by helminth parasites is to manipulate and modulate immunity in order to defuse immune defenses. Multiple mediators (i.e. proteins, glycans, lipids and nucleic acids) intervene in this process (Coakley et al., 2016). The presence of miRNAs and other small RNAs packed in EVs suggests that they might also be involved in this regulation.

Reasoning in a dose-effect manner, we studied the possible effects of the most abundant EV miRNAs, by predicting targeted sites in the 3’ UTR of host genes. Several essential pathways like TGF-β, MAPK, PI3K/AKT, Wnt signaling and particular pathways related to the establishment of an immune response were highlighted as putative enriched targets (**Figure 5**), suggesting a possible role in modulating host’s responses. These results are consistent with previous reports in helminths (reviewed by (Arora et al., 2017)) and with more recent publications that highlight several signaling pathways (Wnt, MAPK, TNF and NOD-like) as potential targets of *F. hepatica*’s miRNAs (Ovchinnikov et al., 2020; Tran et al., 2021). In this sense, fhe-miR-125b (named miR-10-P2a here) was found as the most abundant parasitic miRNA within peritoneal macrophages of infected mice (Tran et al., 2021). Similarly, parasite-derived sma-miR-10 and sma-bantam were found in cells isolated from Peyers patches and mesenteric lymph nodes of *S. mansoni* infected mice (Meningher et al., 2020). A reduction of MAP3K7 expression and reduced NF-κB activity by sma-miR-10 was observed *in vitro*, suggesting a mechanism for the downregulation of the Th2 response. Additionally, sja-bantam and sja-miR-125b were found to induce Th2 key mediator, TNF-α in macrophages *in vitro* and *in vivo*, facilitating parasite development and egg deposition (Liu et al., 2019). Furthermore, parasite-derived let-7 has been predicted to regulate the Wnt signaling pathway and T/B cell activation in mouse genes, and to induce a Th2 immune response in macrophages treated with miRNA mimics in cestodes (Ancarola et al., 2017; Wang et al., 2021). Th2 modulation is a common theme in parasite immune-evasion (Coakley et al., 2016). Interestingly, the miR-10, bantam and let-7 family members are consistently amongst the most abundant miRNA signatures in EVs secreted by adult trematodes (**Table S5**), suggesting that their enrichment is not produced by chance, therefore, constituting central players in modulating host responses to the parasite.

### EVs Pack Homodimeric tRDFs That Could Resist Exonuclease Degradation

From early on, tRNA-derived fragments (tRDF) have been claimed to be involved in the regulation of cellular stress, since their emergence was upregulated in cells exposed to different types of stress stimuli. These tRDFs have been linked to diverse processes including translational regulation, proliferation, apoptosis, stress-granule formation, mRNA stabilization, transposon expression, ribosome biogenesis and the inheritance of acquired traits (Tosar and Cayota, 2020).

Our analysis highlighted that a diverse set of fragments derived from a restricted set of tRNAs were present in different stages related to the mammalian invasion, and in the extracellular vesicles of the adult worm. While 5’ HF from almost every tRNA precursor can be found, their abundance is skewed with those derived from tRNA^Gly_GCC^ as the prominent in all the stages analyzed, and only four others (namely Lys_TTT, Cys_GCA, Asp_GTC, and Met_CAT) highly represented in somatic or EVs samples (**Figure 6B**). Similarly, shorter tRNA fragments detected showed a marked skew in representation, with those derived of tRNA^Gly_GCC^ again as one of the predominant classes. Interestingly, we detected the presence of a novel type of fragment generated by preferential cleavage at the T loop of the precursor, derived mainly from tRNA ^Gln_CTG^ that were highly abundant in EVs. Moreover, most of the tRNA precursors highlighted here were also abundant along the life cycle of *F. gigantica* (Hu et al., 2021) or in the EVs of *S. mansoni* (Nowacki et al., 2015). A marked skew in the representativity of the tRNA derived molecules contained in EVs have also been reported in several species of nematodes, trematodes and cestodes was also evident (Nowacki et al., 2015; Quintana et al., 2019; Wang et al., 2020; Cucher et al., 2021). This could be a consequence of sub-representation due to the difficulties imposed by modified bases in the amplification/sequencing process (Potapov et al., 2018). Novel methods relying on different enzymes and adapters are being developed to overcome this technical restriction, allowing the capture and sequencing of RNAs containing modified bases (Qin et al., 2016; Xu et al., 2019; Shi et al., 2021; Tosar et al., 2021). Surely these methods would provide in the future a more complex picture of the tRNA derived fragments. But so far, we could analyze and compare those detected by the more traditional small RNA sequencing methodologies in different species. On the other hand, the repeated enrichment of certain fragments across stages and species might correspond to their increased resistance to degradation. It has been reported that human tRNA fragments form homodimers and heterodimers that hide the 3’ends and show resistance to degradation by exonucleases (Tosar et al., 2018). We show that the three abundant tRNA fragments found in *F. hepatica* could form homodimers with hidden 3’ ends, similar to those described in humans. While in humans the tRDFs with dimerization capacity were found to be more abundant in the extracellular space, not associated to the EV fraction (Tosar et al., 2018), we found them in EVs, but we did not investigate so far their presence as free circulating tRDFs.

The roles of tRDFs are still poorly understood in platyhelminthes, however it was recently reported that they may be involved in the regeneration of planaria (Cao et al., 2020). Interestingly, in these organisms, Piwi proteins and Ago1, but not Dicer or Ago2, were associated with the generation and/or function of 5’tHFs and 5’tRFs, respectively (Lakshmanan et al., 2021). However, the absence of Piwi genes in all Neodermatans implies that, at least the biogenesis of 5’tHFs could be different in trematodes and cestodes (Fontenla et al., 2017; Fontenla et al., 2021) to those described in free-living flatworms. In any case, many questions related to the mechanisms of generation and the possible roles of tRDFs remain open.

### VtRNAs Are Enriched in the Secreted Fraction

Vault ribonucleoproteins are large hollow barrel-like shape particles in the cytoplasm of many eukaryotic cells. Although little is known yet on their function, they have been involved in key regulatory roles including autophagy, apoptosis and modulation of gene expression [reviewed by Frascotti et al., (2021)]. Even more, recent evidence has shown that human vtRNA1-1 could guide sequence-specific cleavage of a complementary target RNA (Frascotti et al., 2021).

Within flatworms vtRNAs have only been identified previously in *Macrostomum lignano* (https://rfam.xfam.org/), and although they were expected to be present in *S. mansoni* since a homolog of the Major Vault Protein (MVP) is coded in its genome, no clear sequences were identified (Copeland et al., 2009). Proteomic analysis of *F. hepatica* EVs detected the presence of MVPs, raising the question if the corresponding vtRNAs were also present. Our approach based on a relaxed homology search with a short but most conserved region of phylogenetically proximal lophotrochozoan vtRNAs, followed by a thorough manual curation was key to detect 5 putative vtRNAs in *F. hepatica*. The identified sequences have short, conserved regions similar to other vtRNAs and can be folded producing similar structures, suggesting they are bona-fide vtRNAs. Furthermore, a rapid analysis of available transcriptomic data provided evidence of their expression in diverse samples and stages. Taken together, these results suggest that a similar approach can now be followed to seek their presence in other trematodes. Although the functional role of vtRNAs from *F. hepatica* is unknown, their selective packaging into fluke EVs suggests that they could participate in host-parasite interactions.

## Conclusions

We have analyzed the profiles of expression of the small RNAs complement in three intra-mammalian stages of the life cycle of *F. hepatica*. We consistently detected the presence of miRNAs, tRNA derived molecules and vtRNAs in all the samples. This is the first description of the still little known vtRNAs in trematodes. Interestingly the same three types of RNAs were present in EVs generated by the adult worms, stressing their putative role in crosstalk to the host.

Within miRNAs, those more abundant in EVs correspond to conserved families predicted to target several host signaling pathways. Interestingly, this seems to be a common theme in helminths, with increasing reports of uptake of parasite-derived miRNAs by host cells, and *in vitro* evidence of downregulation of host genes associated with the immune response (Arora et al., 2017). Additionally, a skewed population of tRNA fragments were detected in all the stages analyzed. We described here a new class of tRNA fragment, produced by the cleavage at the T-loop particularly abundant in the EV fraction. The most abundant tRNA fragments of the EV fraction can form stable homodimeric structures that might explain their increased stability. The roles of these tRNA fragments in regulation of *F. hepatica* and/or in the interaction with the host are speculative and await further validation.

Indeed, further experimental approaches are needed to understand the roles of all these small RNA classes; their combined presence in EVs suggests a concerted action in the interaction and modulation of the host responses, that deserves to be investigated.

## Data Availability Statement

The original contributions presented in the study are publicly available in NCBI under accession number PRJNA782636.

## Author Contributions

Conceptualization: SF and JT. Samples preparation and sequencing: ET and MR. Analysis: SF and ML. Drafting the manuscript: SF, MR, MD, and JT. All authors contributed to the article and approved the submitted version.

## Funding

The research was founded by CSIC grant 521_2019 and BBSRC grant BB/L019612/1. SF, MD, and JT are part of the National Researchers System (SNI). JT is part of the Basic Sciences Development Program (PEDECIBA).

## Conflict of Interest

The authors declare that the research was conducted in the absence of any commercial or financial relationships that could be construed as a potential conflict of interest.

## Publisher’s Note

All claims expressed in this article are solely those of the authors and do not necessarily represent those of their affiliated organizations, or those of the publisher, the editors and the reviewers. Any product that may be evaluated in this article, or claim that may be made by its manufacturer, is not guaranteed or endorsed by the publisher.
