# Supplementary figures and images for: Role of Fasciola hepatica Small RNAs in the Interaction With the Mammalian Host
Source: Front Cell Infect Microbiol. 2022 Jan 20;11:812141. doi: 10.3389/fcimb.2021.812141 (PMC8824774; doi:10.3389/fcimb.2021.812141)

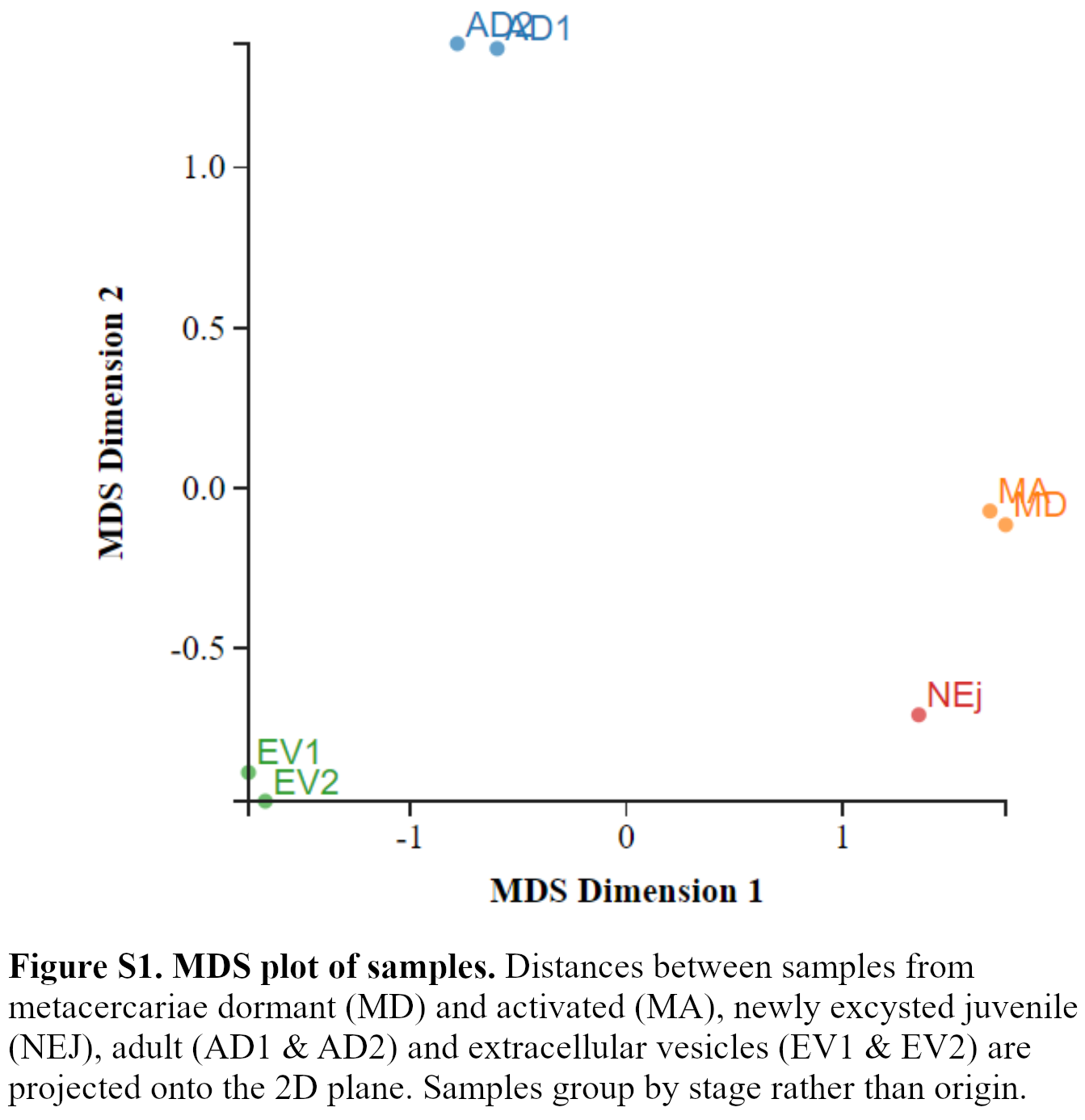

Supplement: Supplementary file 1 [file Image_1.tif]

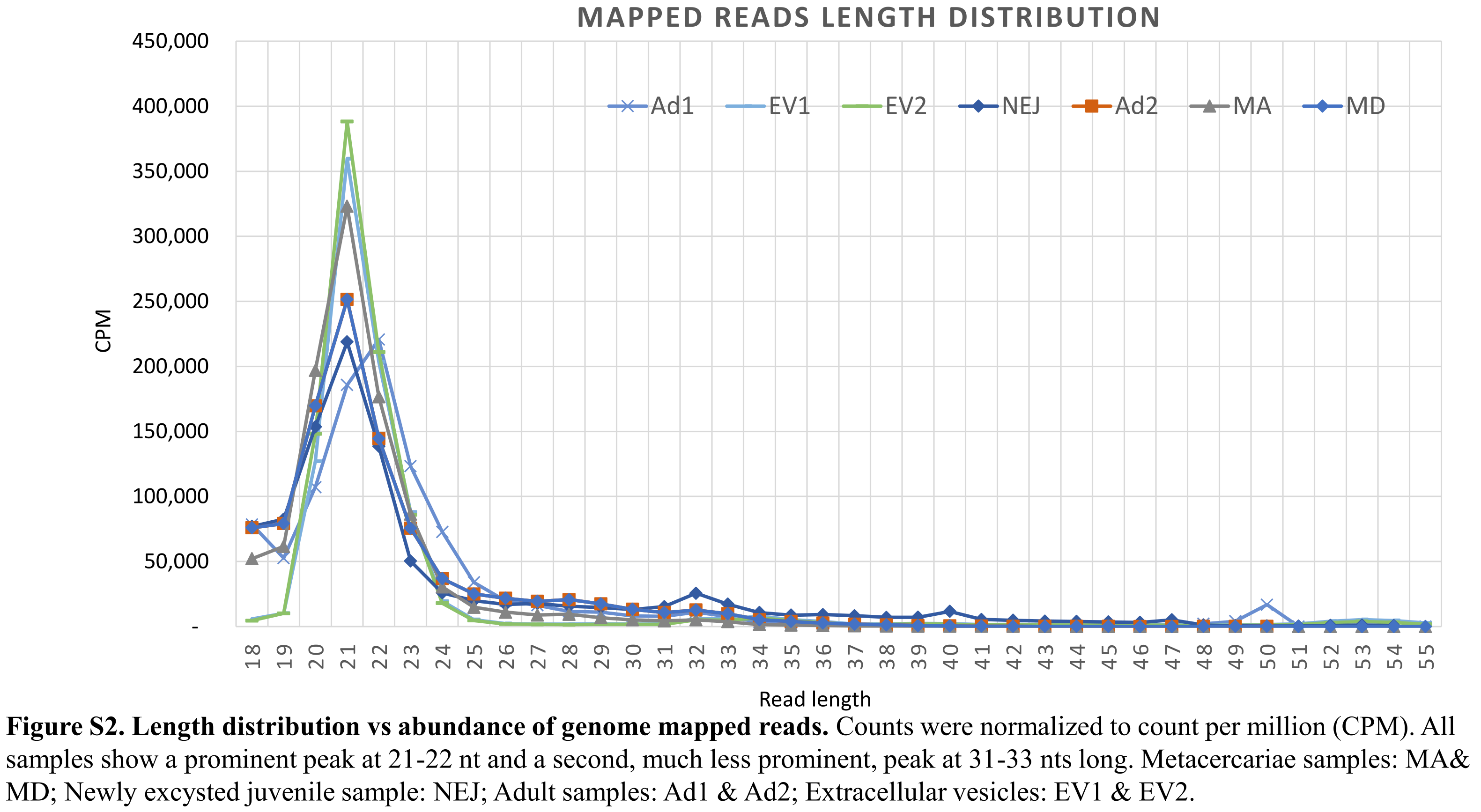

Supplement: Supplementary file 2 [file Image_2.tif]

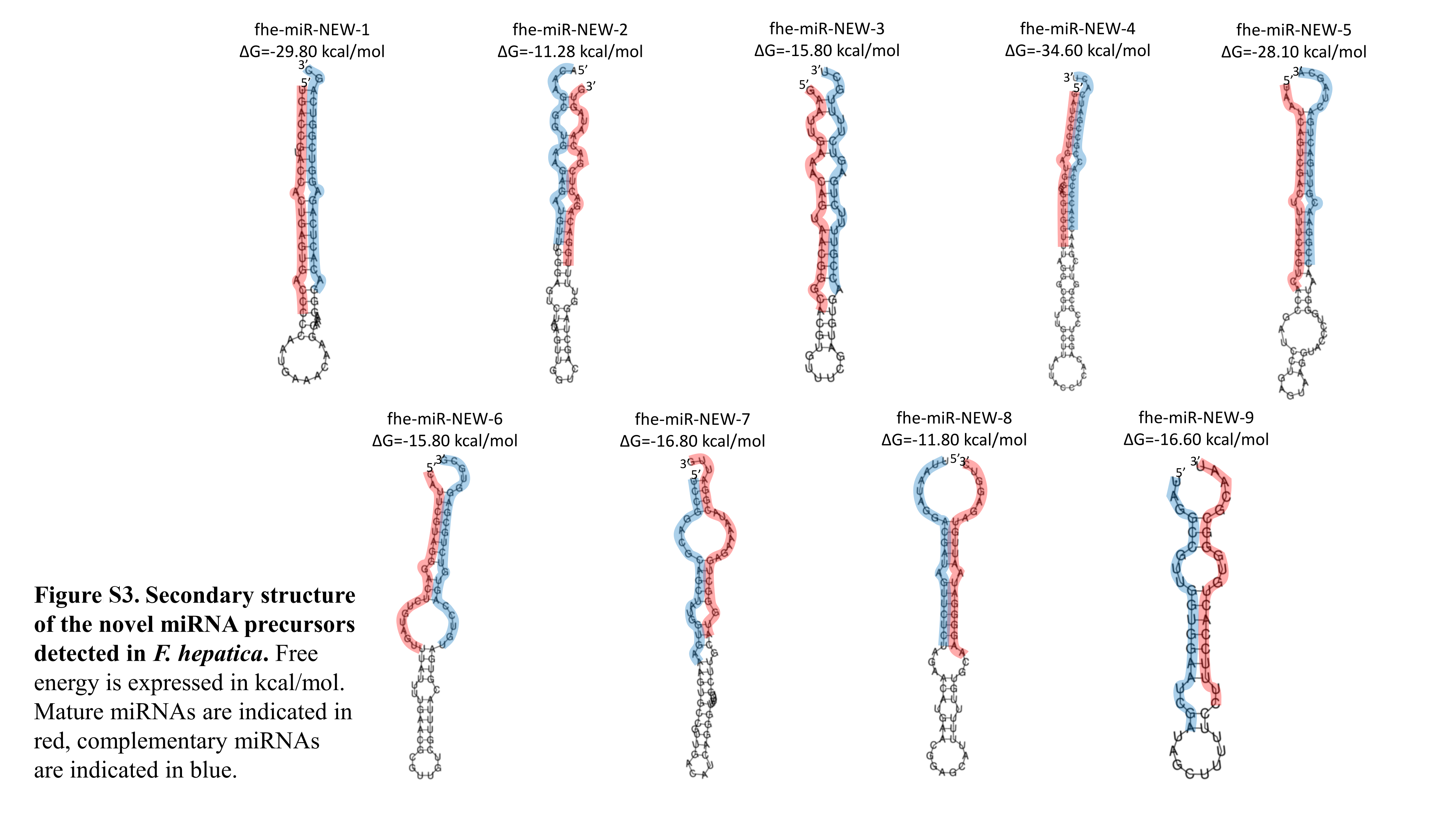

Supplement: Supplementary file 3 [file Image_3.tif]

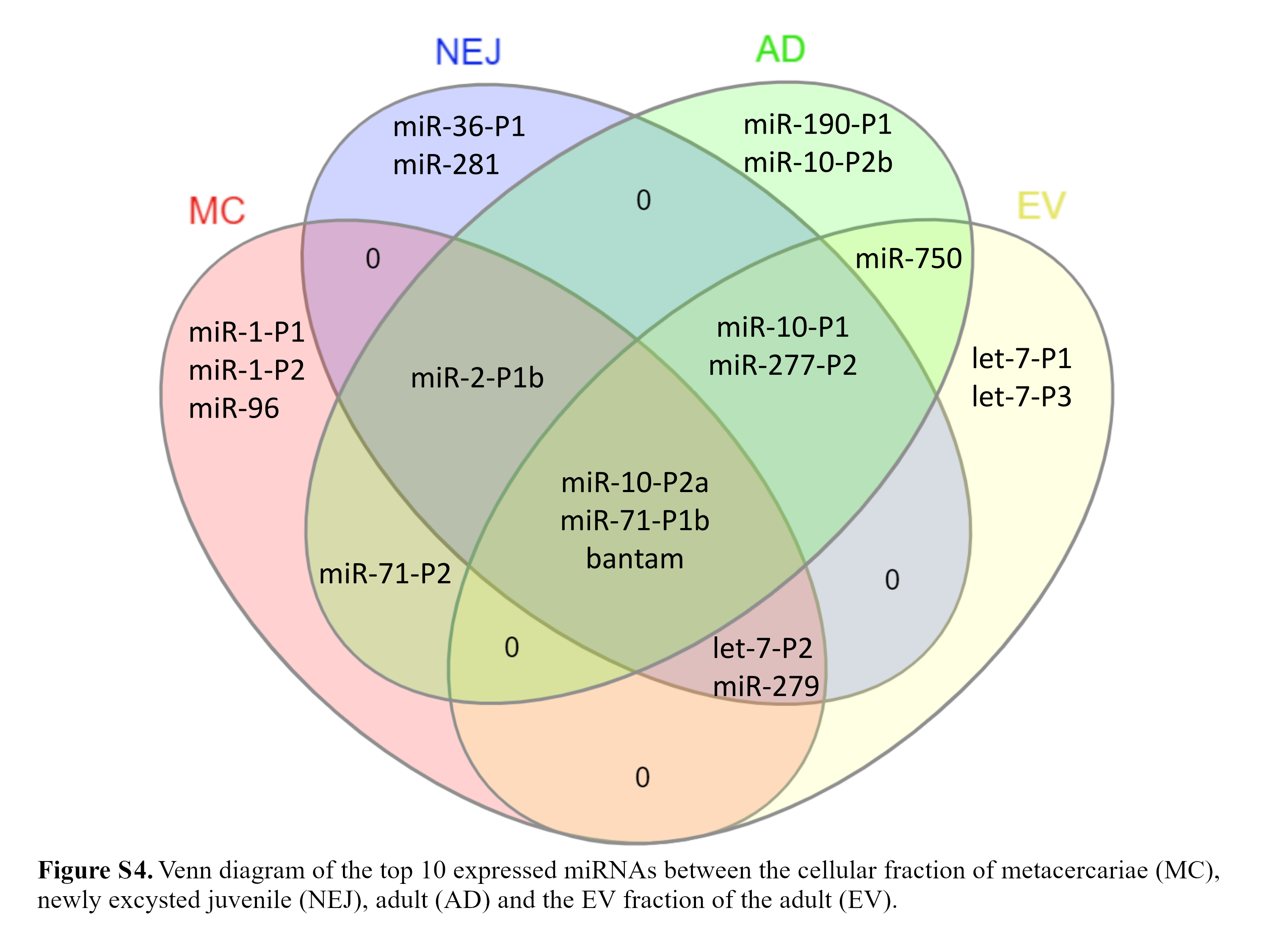

Supplement: Supplementary file 4 [file Image_4.tif]

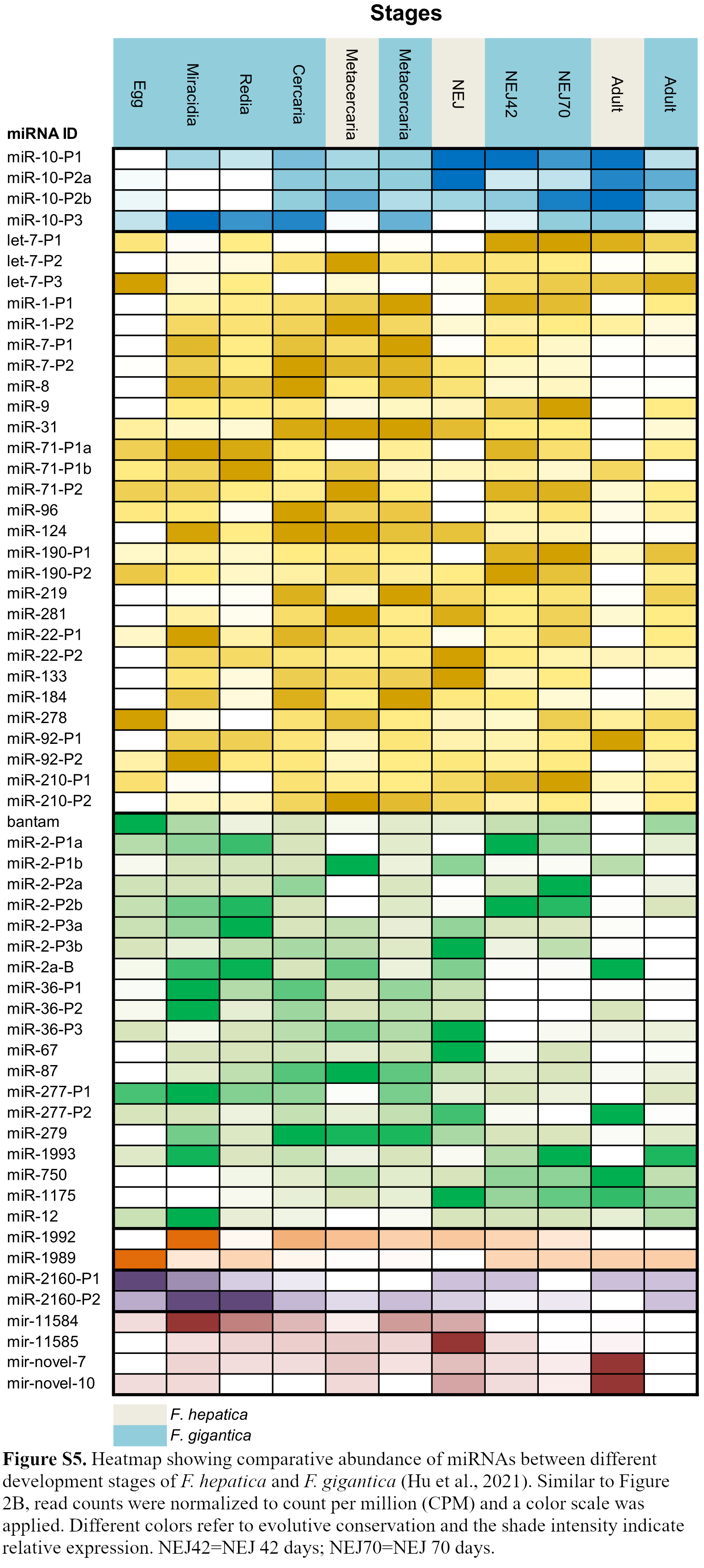

Supplement: Supplementary file 5 [file Image_5.tif]

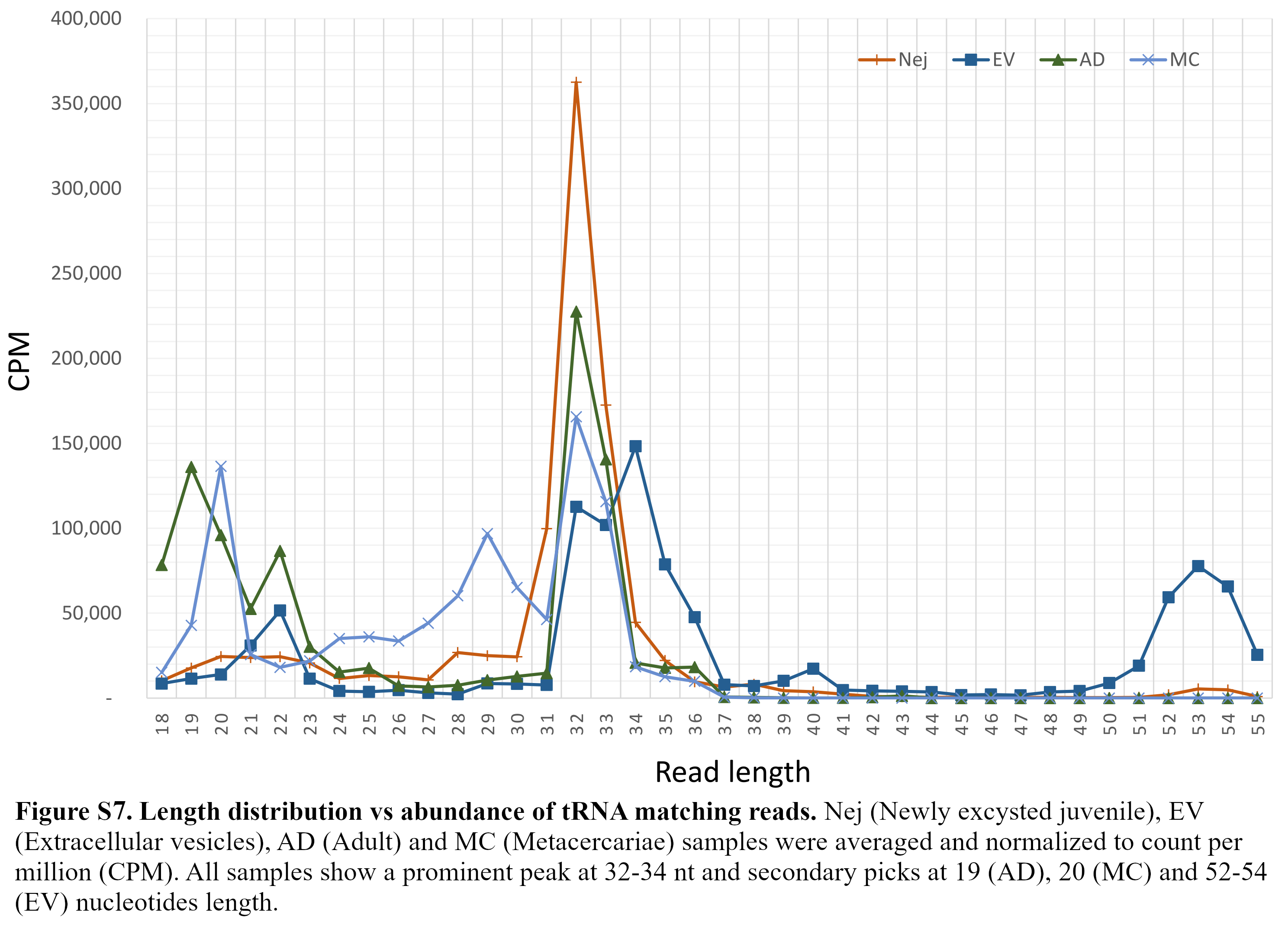

Supplement: Supplementary file 7 [file Image_7.tif]

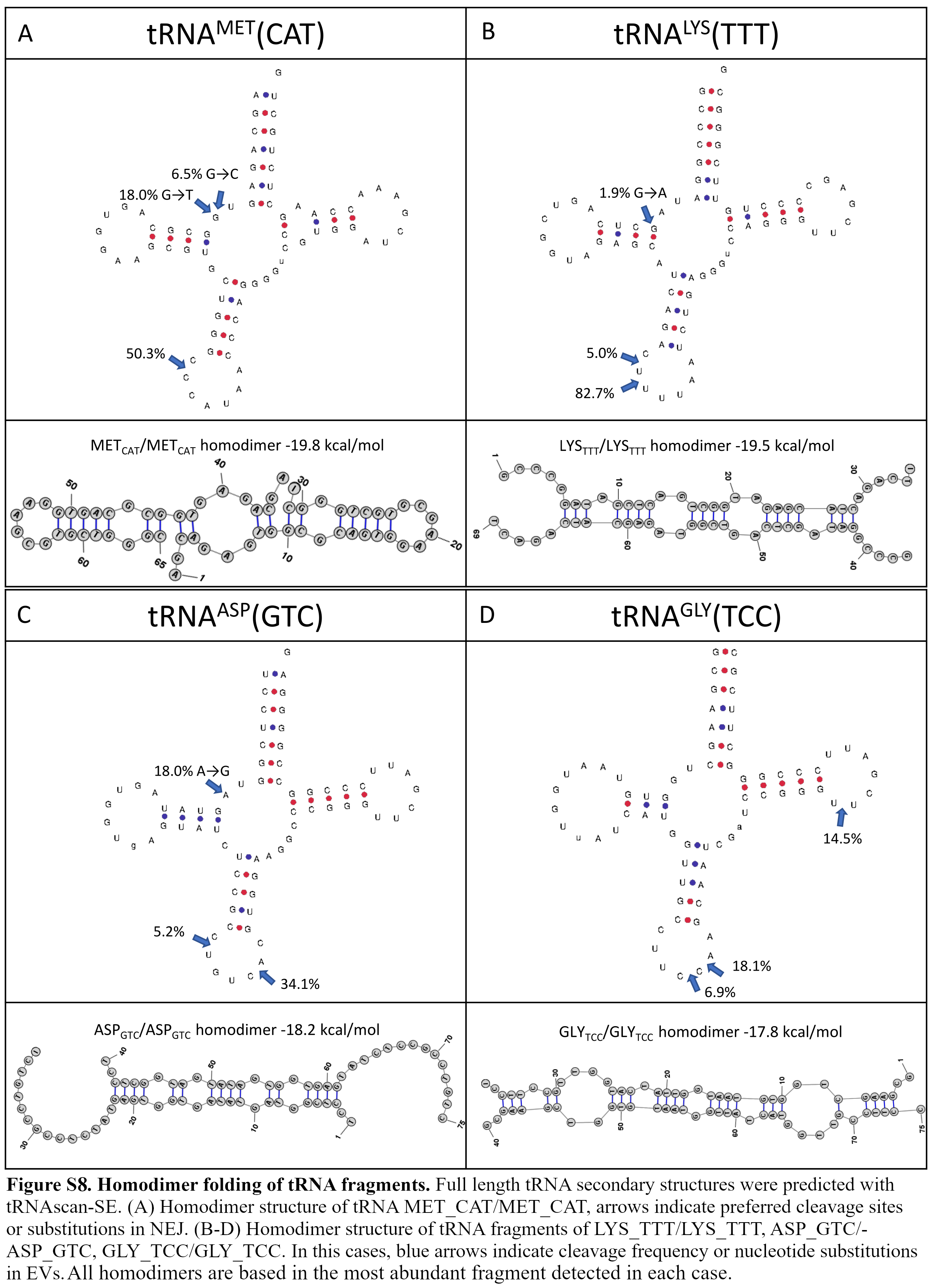

Supplement: Supplementary file 8 [file Image_8.tif]

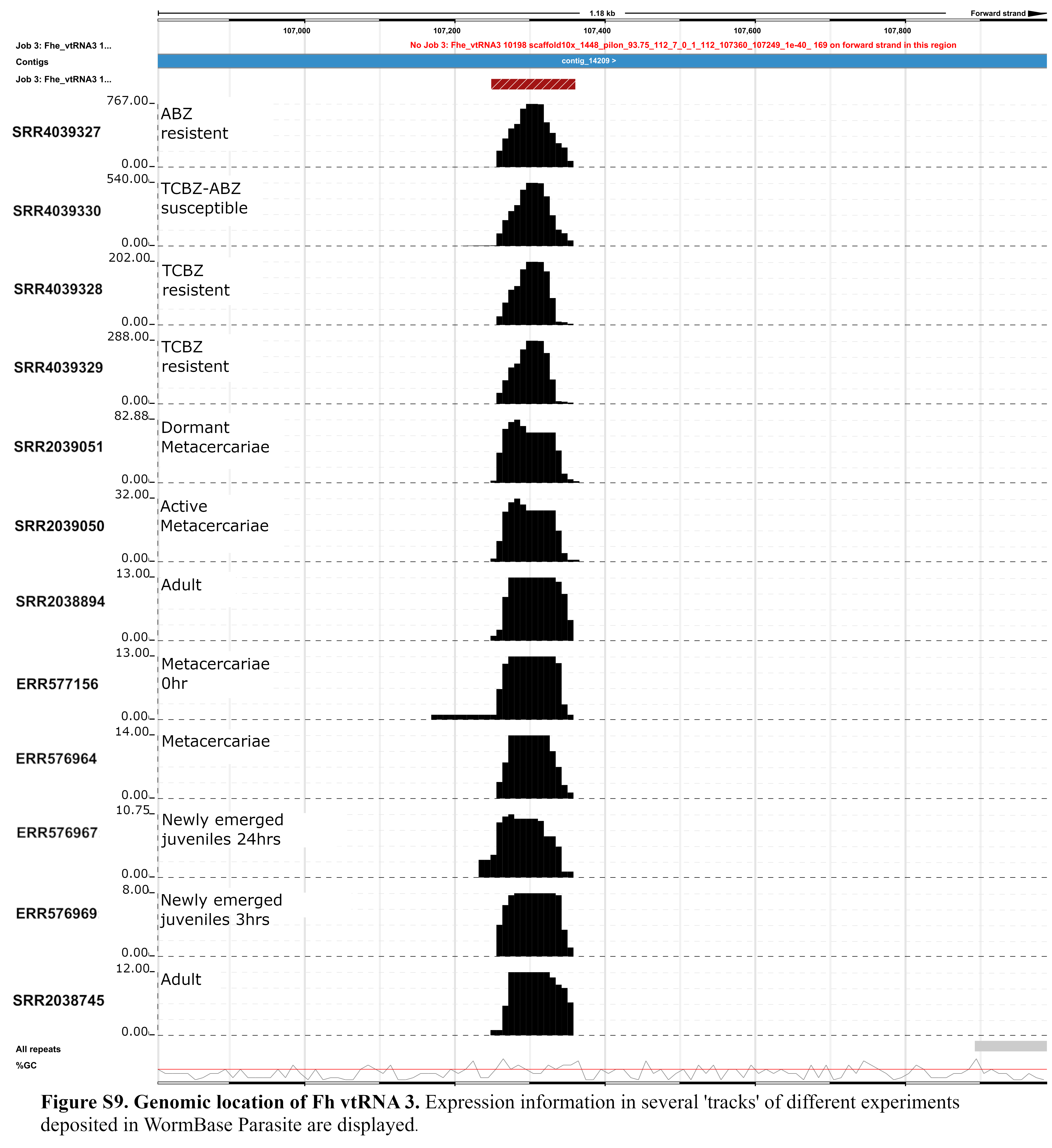

Supplement: Supplementary file 9 [file Image_9.tif]
