# Supplementary material for: Role of Fasciola hepatica Small RNAs in the Interaction With the Mammalian Host
Source: Front Cell Infect Microbiol. 2022 Jan 20;11:812141. doi: 10.3389/fcimb.2021.812141 (PMC8824774; doi:10.3389/fcimb.2021.812141)
Supplement: Supplementary file 6 [file Image_6.pdf]

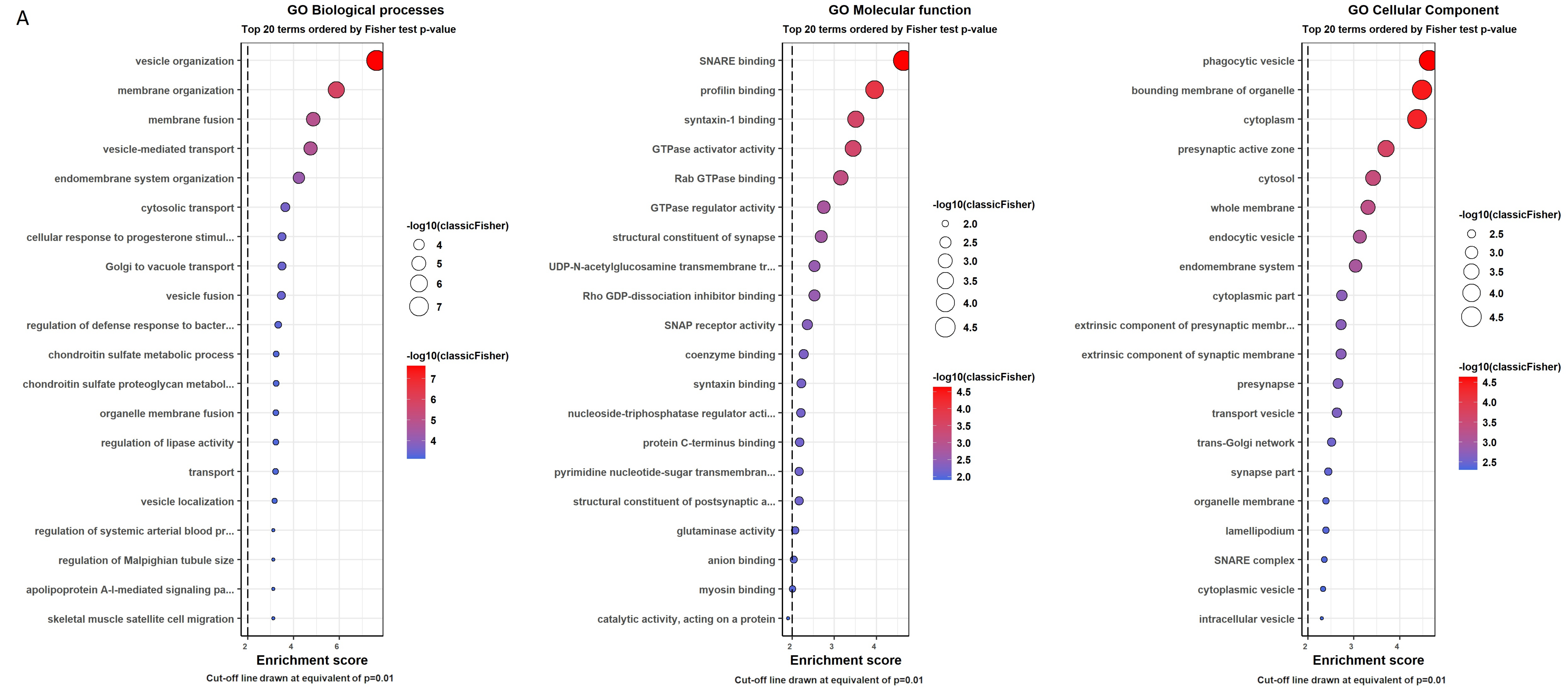

Figure S6A. Top 20 GO terms enriched in the target genes of miRNAs in cluster 1 (upregulated in metacercariae) of Figure 3A. Enrichment score was calculated as -log of p-value, terms with a score ≥ 2 (dashed line) were considered significant.

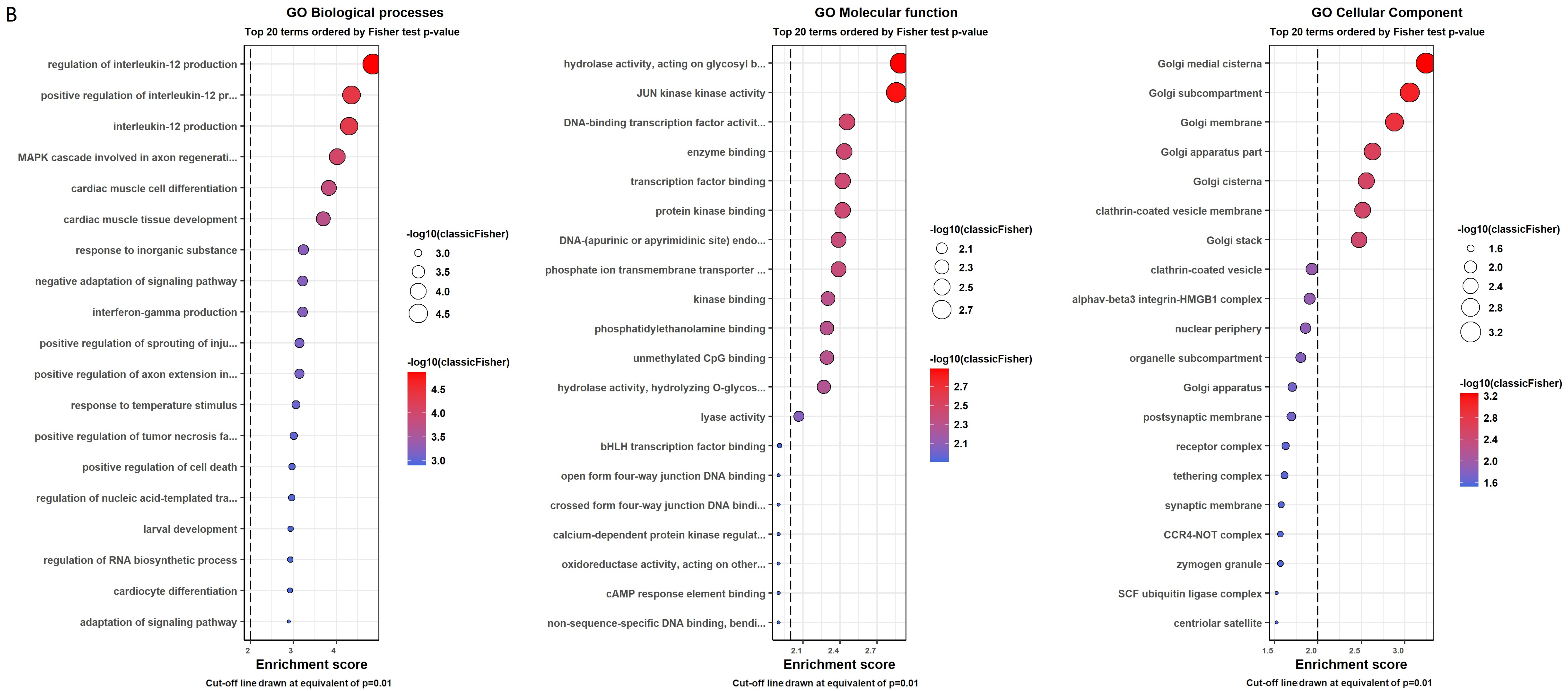

Figure S6B. Top 20 GO terms enriched in the target genes of miRNAs in cluster 2 (downregulated in metacercariae) of Figure 3A. Enrichment score was calculated as -log of p-value, terms with a score ≥ 2 (dashed line) were considered significant.

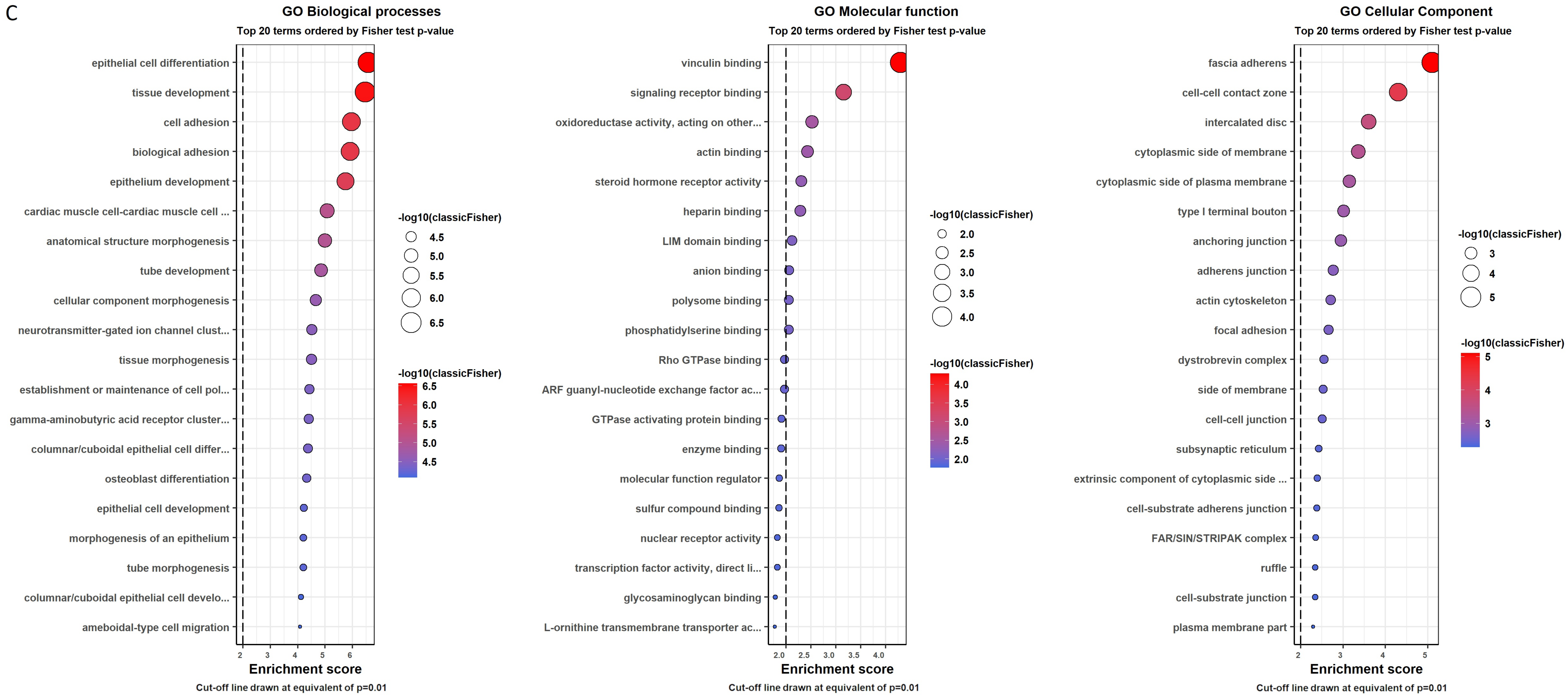

Figure S6C. Top 20 GO terms enriched in the target genes of miRNAs in cluster 3 (upregulated in adult) of Figure 3A. Enrichment score was calculated as -log of p-value, terms with a score ≥ 2 (dashed line) were considered significant.

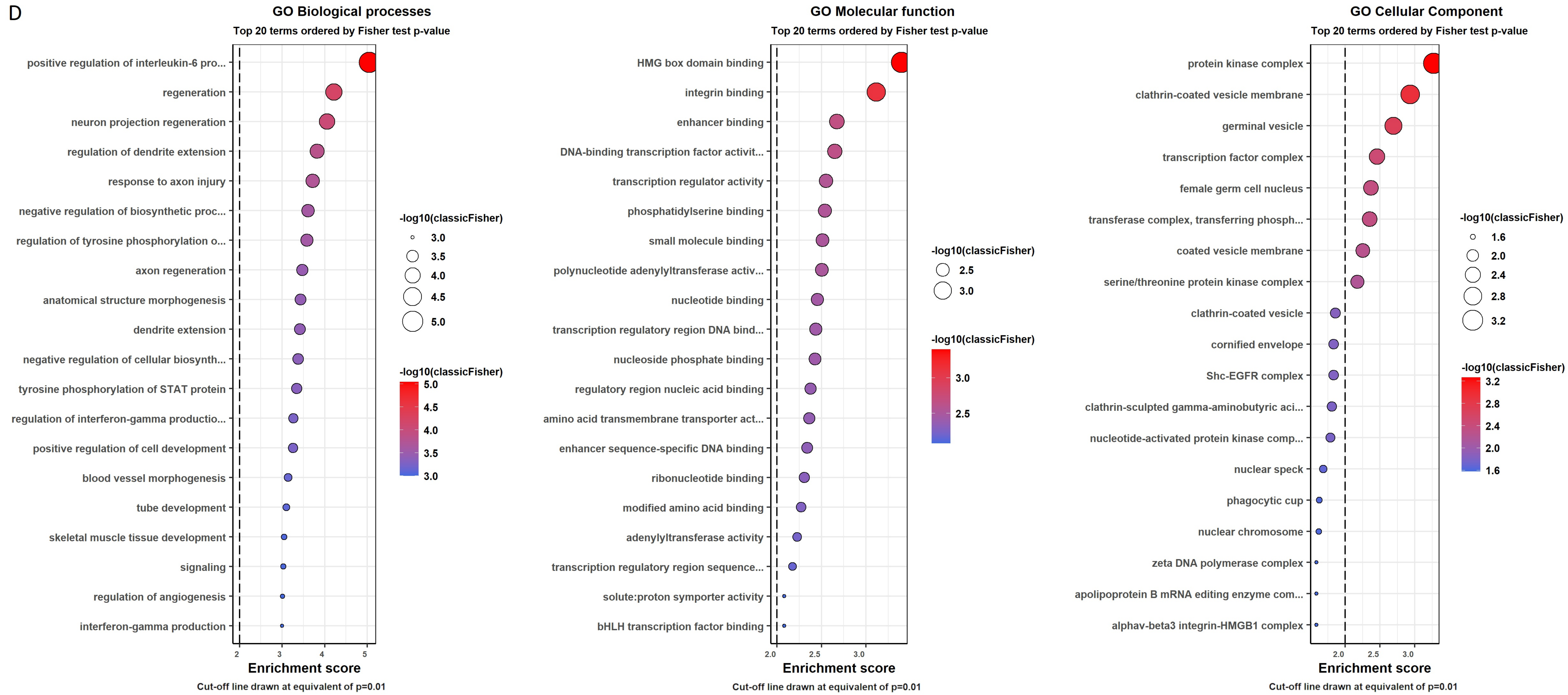

Figure S6D. Top 20 GO terms enriched in the target genes of miRNAs in cluster 4 (upregulated in NEJ) of Figure 3A. Enrichment score was calculated as -log of p-value, terms with a score ≥ 2 (dashed line) were considered significant.

E

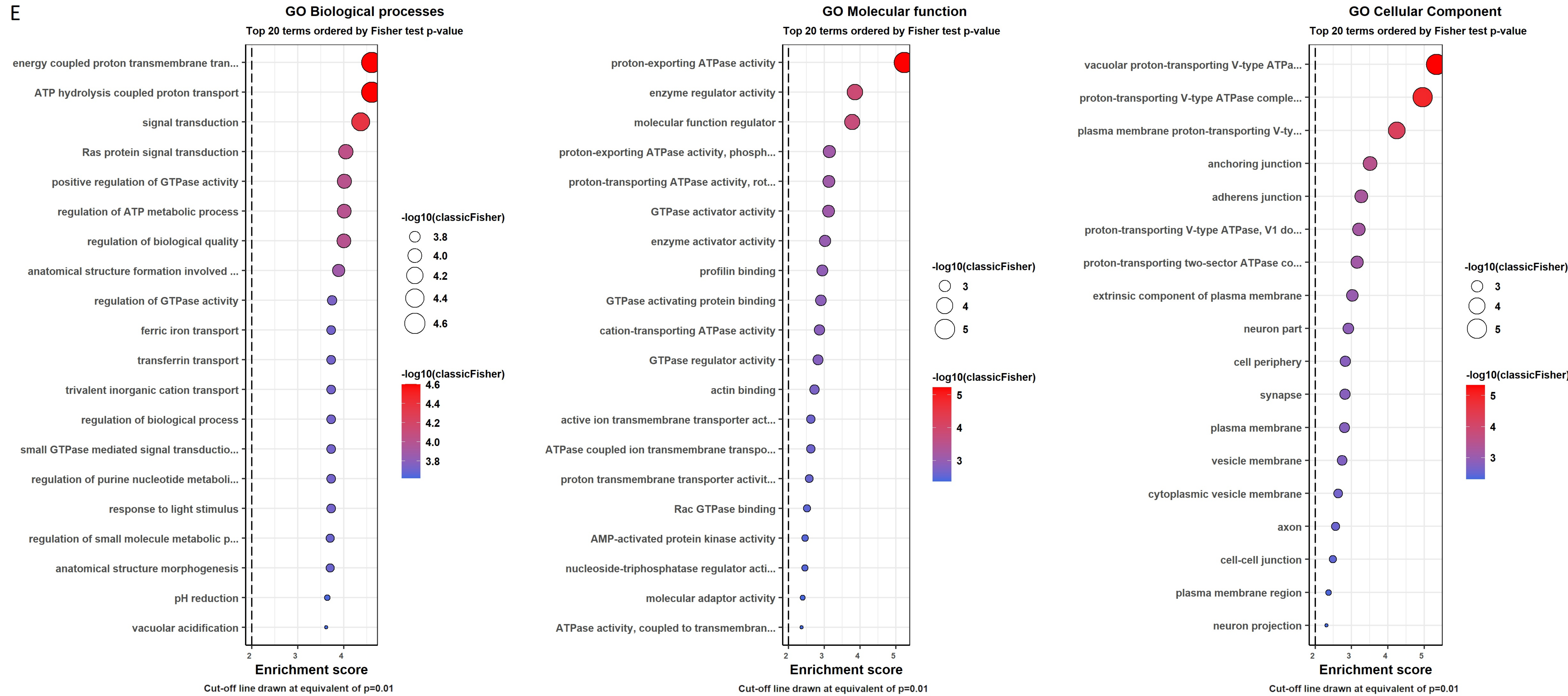

Figure S6E. Top 20 GO terms enriched in the target genes of miRNAs in cluster 5 (downregulated in adult) of Figure 3A. Enrichment score was calculated as -log of p-value, terms with a score ≥ 2 (dashed line) were considered significant.
